# Increased apoptotic sensitivity of glioblastoma enables therapeutic targeting by BH3-mimetics

**DOI:** 10.1038/s41418-022-01001-3

**Published:** 2022-04-26

**Authors:** Anna L. Koessinger, Catherine Cloix, Dominik Koessinger, Dieter Henrik Heiland, Florian J. Bock, Karen Strathdee, Kevin Kinch, Laura Martínez-Escardó, Nikki R. Paul, Colin Nixon, Gaurav Malviya, Mark R. Jackson, Kirsteen J. Campbell, Katrina Stevenson, Sandeep Davis, Yassmin Elmasry, Asma Ahmed, Jim O’Prey, Gabriel Ichim, Oliver Schnell, William Stewart, Karen Blyth, Kevin M. Ryan, Anthony J. Chalmers, Jim C. Norman, Stephen W. G. Tait

**Affiliations:** 1 Cancer Research UK Beatson Institute, Glasgow, G61 1BD UK; 2 Institute of Cancer Sciences, University of Glasgow, Glasgow, G61 1BD UK; 3 Department of Neurosurgery, Medical Centre, University of Freiburg, Breisacher Straße 64, 79106 Freiburg, Germany; 4 Department of Radiotherapy (MAASTRO), GROW-School for Oncology and Developmental Biology, Maastricht University, 6229 ER Maastricht, The Netherlands; 5 Department of Neuropathology, Queen Elizabeth University Hospital and Institute of Neuroscience and Psychology, University of Glasgow, Glasgow, UK; 6 Cancer Research Centre of Lyon (CRCL) INSERM 1052, CNRS 5286 Lyon, France

**Keywords:** Cancer genetics, Cell biology

## Abstract

Glioblastoma (GBM) is the most prevalent malignant primary brain tumour in adults. GBM typically has a poor prognosis, mainly due to a lack of effective treatment options leading to tumour persistence or recurrence. We investigated the therapeutic potential of targeting anti-apoptotic BCL-2 proteins in GBM. Levels of anti-apoptotic BCL-xL and MCL-1 were consistently increased in GBM compared with non-malignant cells and tissue. Moreover, we found that relative to their differentiated counterparts, patient-derived GBM stem-like cells also displayed higher expression of anti-apoptotic BCL-2 family members. High anti-apoptotic BCL-xL and MCL-1 expression correlated with heightened susceptibility of GBM to BCL-2 family protein-targeting BH3-mimetics. This is indicative of increased apoptotic priming. Indeed, GBM displayed an obligate requirement for MCL-1 expression in both tumour development and maintenance. Investigating this apoptotic sensitivity, we found that sequential inhibition of BCL-xL and MCL-1 led to robust anti-tumour responses in vivo, in the absence of overt toxicity. These data demonstrate that BCL-xL and MCL-1 pro-survival function is a fundamental prerequisite for GBM survival that can be therapeutically exploited by BH3-mimetics.

## Introduction

In adults, glioblastoma (GBM) is the most prevalent and malignant primary brain tumour [1, 2]. Despite current multimodal treatment, comprising surgical resection with adjuvant radiotherapy and alkylating chemotherapy, the median survival in newly diagnosed patients remains poor at less than 12 months [3, 4]. Resistance to conventional radio- and chemotherapy primarily emerges from persistent cancer stem cells, a tumourigenic subpopulation of GBM cells, consisting of heterogenous subclones and capable of self-renewal [5, 6]. Therefore, targeting cells with stem-like capabilities is essential to develop effective treatment options and improve patient survival.

Treatment resistance can often be attributed to cells circumventing therapy-induced cell death. Apoptosis is an evolutionarily conserved type of cell death with broad ranging importance in biology [7]. The intrinsic (mitochondrial) pathway of apoptosis is controlled by pro- and anti-apoptotic members of the B cell lymphoma 2 (BCL-2) family that regulate mitochondrial outer membrane integrity [8, 9]. During apoptosis, pro-apoptotic BCL-2 proteins cause mitochondrial outer membrane permeabilisation or MOMP. This leads to the release of mitochondrial intermembrane space proteins, including cytochrome *c*, that activate caspase proteases resulting in apoptotic cell death [8].

Increased anti-apoptotic BCL-2 protein expression has been described in a wide range of solid cancers and is often linked with insensitivity to conventional chemotherapy [10–12]. Recently, a new class of chemotherapeutics called BH3-mimetics have been developed that target pro-survival BCL-2 function, sensitising to cell death [9, 13, 14]. BH3-mimetics have proven to be highly effective in haematologic malignancies [15–17]. For instance, venetoclax (ABT-199), a BCL-2 targeted BH3-mimetic [18], is in clinical use for chronic lymphocytic leukaemia (CLL) [19] and acute myelogenous leukaemia (AML) [20–22]. CLL cells typically express high levels of anti-apoptotic BCL-2 protein [17]. Nevertheless, the high intrinsic apoptotic sensitivity - also called apoptotic priming - of CLL renders it sensitive to venetoclax. For solid cancers, venetoclax is currently being tested in combination with conventional chemotherapeutic agents. The combination of venetoclax and tamoxifen has progressed to early phase clinical trials in patients with estrogen receptor positive (ER+), high BCL-2 expressing breast cancer [23]. Other BH3-mimetics developed to target BCL-xL and MCL-1 have shown promising pre-clinical results in combination with inhibitors of MEK1/2 for solid cancers harbouring oncogenic mutations in the MAPK pathway [24–26]. Other than GBM, CNS-WHO grade 4 astrocytoma carry a mutation of isocitrate dehydrogenase 1 (*IDH1*) [1], which has been linked with increased sensitivity to treatment with BH3-mimetics targeting BCL-xL [27]. Furthermore, previous studies have proposed BCL-xL as a treatment target in combination with ionising radiation [28] and other chemotherapeutics [29] in GBM.

Because tumours retain characteristics of their tissue origins, brain-derived glial cancers exhibit defined cellular hierarchies found in brain development and homeostasis [30–32]. During central nervous system development, anti-apoptotic BCL-2 family proteins play a pivotal role in promoting cell survival [33, 34] while with adulthood the brain becomes refractory to apoptosis [35]. Given this important role in cell survival, we hypothesised that GBM, while phenocopying the developing brain, might display similar anti-apoptotic survival dependencies. Indeed, we found increased levels of the major pro-survival proteins in GBM, specifically within the stem-cell enriched population. Interestingly, high BCL-xL and MCL-1 expression correlates with increased apoptotic sensitivity, demonstrating that GBM stem-like cells are primed for apoptosis. Exploiting this, we found that sequential dosing of BCL-xL and MCL-1 targeting BH3-mimetics enables effective treatment responses both, in vitro and in vivo. This could offer a therapeutically tractable approach for patients with GBM.

## Results

### High anti-apoptotic BCL-2 family protein expression correlates with increased BH3-mimetic sensitivity in GBM

**Fig. 1:**
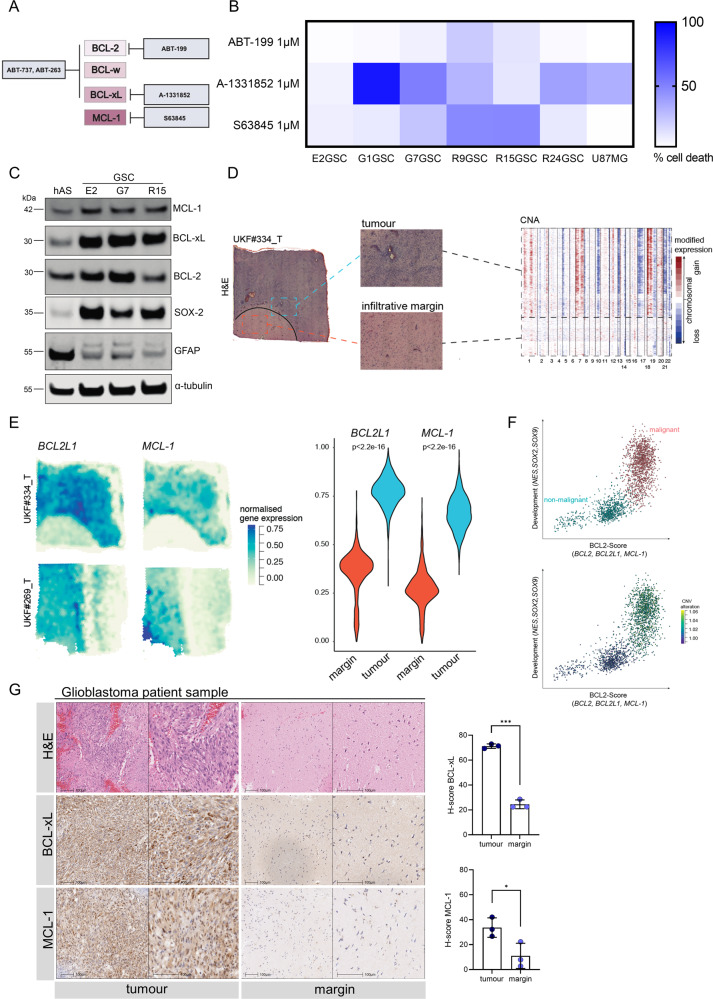
High anti-apoptotic BCL-2 family protein expression correlates with increased BH3-mimetic sensitivity in GBM. **A** Schematic overview of BH3-mimetic drugs used and their respective targets (**B**) Panel of six GSC cell lines and the human GBM cell line U87MG were treated with indicated drugs for 24 to 48 h and analysed for cell viability using an IncuCyte imager and SYTOX Green exclusion. Results are presented as heatmap. Percentage cell death was calculated by normalising against maximal cell death (treatment with 1 µM Actinomycin D, 10 µM ABT-737 and 1 µM S6384), *n* = 3 independent experiments per cell line. **C** Immunoblot of BCL-2 family proteins, cell-line specific neural stem cell marker SOX2 and astrocyte lineage differentiation marker GFAP in human astrocytes (hAS) and patient-derived GSC. α-tubulin served as loading control. Representative image from *n* = 3 independent experiments. **D** Haematoxylin and eosin staining (H&E) of the GBM sample (#UKF334_T, left). On the right side, heatmap of inferred copy-number alterations demonstrate the tumour core (high CNA), and margin regions (low CNA). **E** Surface plot of spatially resolved expression of *BCL2L1* and *MCL-1* gene expression analysis (left) of two examples. On the right side, a violin plot of the expression of *BCL2L1* and *MCL-1* across six patients with core and margins. Statistical analysis was performed by Mann–Whitney-U-test and *p* value corrected by Benjamini Hochberg. **F** Correlation of gene expression of the pooled *BCL2* genes (*BCL2, BCL2L1* and *MCL-1*) and the neural stem cells markers (*NES, SOX2* and *SOX9*) based on single-cell sequencing of GBM cells from six GBM specimens. **G** Matched tumour and margin specimens were obtained from three patients diagnosed with GBM and stained for H&E (upper row), BCL-xL (middle row) and MCL-1 IHC (lower row). Representative images of one case shown. H-score (analysis of intracellular BCL-xL and MCL-1 expression) was determined using automated analysis with Halo. Error bars represent mean ± SD (***p = 0.0003, *p = 0.0404) Welch’s test. Further representative images in lower magnifications are shown in Supplementary Fig. 1C.

Cancer stem cells are proposed to give rise to GBM and contribute to therapeutic resistance [30]. We therefore sought to assess the apoptotic sensitivity of GBM stem-like cells (GSC) by treating them with BH3-mimetics with selectivity for BCL-2, BCL-xL and MCL-1. For this purpose, we used a panel of patient-derived *IDHwt* GSC, cultured under conditions to maintain their tumour specific phenotype and stem cell properties [36, 37]. Cell viability was measured using IncuCyte live cell imaging and SYTOX Green exclusion. Importantly, three cell lines (G1, G7 and R24 GSC) were sensitive to A-1331852, a selective BCL-xL antagonist [38], whereas two cell lines (R9 and R15 GSC) displayed sensitivity to S63845, a potent and selective MCL-1 inhibitor [39] (Fig. 1A, B). Moreover, the commonly used GBM cell line U87MG displayed increased sensitivity to BCL-xL inhibition when cultured under stem cell enriching conditions. One cell line (E2 GSC) was resistant to all single agent treatments. Treatment with venetoclax (ABT-199), a BCL-2 specific inhibitor, induced no more than 26% cell death in any GSC and therefore was comparably inefficient. Collectively, these data show that the majority of tested GSC display a survival dependence on anti-apoptotic BCL-2 family function.

We next used immunoblotting to determine if the individual apoptotic sensitivity of the patient-derived GSC corresponded to anti-apoptotic BCL-2 protein expression. In comparison to human astrocytes, all GSC exhibited higher expression of BCL-xL and MCL-1 as well as partially higher expression of BCL-2 (Fig. 1C), but no conclusive correlation could be drawn from individual protein abundance with sensitivity for BH3-mimetics. As expected, GSC expressed higher levels of neural stem cell marker SOX2 [40], while cell lineage specific GFAP was predominantly found in astrocytes [41].

We next sought to confirm our findings of GSC associated anti-apoptotic BCL-2 family expression patterns in GBM patient tumours. To address this question, we analysed spatially resolved transcriptomic data from the “Freiburg Atlas of Spatial Biology”. Samples were segmented in core and margin in accordance with the Ivy-gap classification system [42]. The analysis revealed that *BCL2L1* (BCL-xL) and *MCL-1* were exclusively expressed in tumour regions marked by the hallmark chromosomal alterations, namely gain in chromosome 7 and loss of chromosome 10 (Fig. 1D, E). Further characterisation by matched single cell RNA-sequencing of the same tumours revealed that *BCL-xL* and *MCL-1* expression correlated with expression of neural stem cell markers *NES, SOX2* and *SOX9* and the degree of malignancy within the tumours (CNA gain chromosome 7) (Fig. 1F), independently of cell cycle (Supplementary Fig. 1A). Applying the Neftel classification [43], we found *BCL-xL* and *MCL-1* expression to be independent of transcriptional GBM subgroups (Supplementary Fig. 1B), further emphasising the general applicability of our findings.

Additionally, immunohistochemistry (IHC) of matched tumour cores and predominantly non-tumorous margins of three patients confirmed increased BCL-xL and MCL-1 expression within the tumour cores compared to the margins (Fig. 1G; Supplementary Fig. 1C). Finally, we determined *BCL-xL* and *MCL-1* mRNA expression in different glioma subtypes and normal brain tissue using the publicly available REMBRANDT database. In line with our analyses, *BCL-xL* and *MCL-1* mRNA levels were higher expressed in GBM compared to lower grade gliomas and normal brain tissue (Supplementary Fig. 1D).

As the therapeutic standard of care for GBM patients consists of radio- and chemotherapy with temozolomide, we combined BH3-mimetics with each treatment. Interestingly, in a selection of GSC, we could neither detect a radiosensitisation by navitoclax (ABT-737), an inhibitor of BCL-xL, BCL-2 and BCL-w [13], nor any combinatory treatment effect for A-1331852 or S63845 with temozolomide (Supplementary Fig. 1E, F).

Taken together, our data demonstrate specific sensitivities of patient-derived GSC to individual BH3-mimetics and increased expression of anti-apoptotic BCL-2 proteins in both primary GBM tumour tissues and GSCs.

### Anti-apoptotic MCL-1 is required for tumour growth and survival in an orthotopic GBM PDX model

**Fig. 2:**
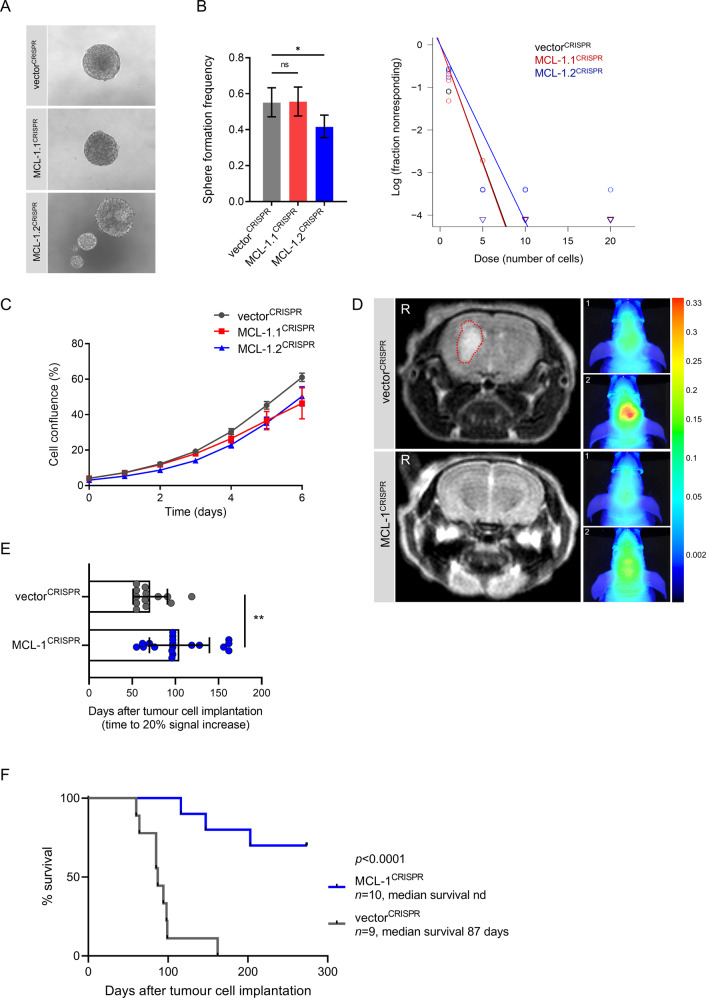
Anti-apoptotic MCL-1 is required for tumour growth and survival in an orthotopic GBM PDX model. **A** Representative images of neurosphere growth from G7 GSC vector^CRISPR^ (upper panel), MCL-1.1^CRISPR^ (middle panel) and MCL-1.2^CRISPR^ (lower panel). **B** Effects on self-renewal in vitro were assessed by an extreme limiting dilution assay (ELDA) for neurosphere formation frequency by G7 GSC vector^CRISPR^ vs. MCL-1.1^CRISPR^ or MCL-1.2^CRISPR^. Error bars represent 95% confidence interval of *n* = 3 independent experiments (nonsignificant (ns) *p* = 0.908, *p = 0.00795, calculated using the ELDA website http://bioinf.wehi.edu.au/software/elda/ [69]). **C** Proliferation assay of indicated cell lines using IncuCyte live cell imaging (percentage cell density over 6 days). Error bars represent mean ± SD from *n* = 3 independent experiments. **D** Representative images of brain MRI scans (tumour indicated by red dashed line) next to corresponding pseudocolour representations of iRFP signal of mice bearing iRFP tagged G7 GSC vector^CRISPR^ (upper panel) and MCL-1.2^CRISPR^ (lower panel) xenografts, respectively. iRFP signal was detected by PEARL scans (700 nm channel) (1) at week 8 and (2) at week 20 (vector^CRISPR^) or week 36 (MCL-1^CRISPR^) post injection. **E** Quantification of time to 20% iRFP signal increase of G7 vector^CRISPR^ *n* = 13 vs. G7 MCL-1^CRISPR^ tumours *n* = 19, compared to four weeks post injection (baseline signal). Error bars represent mean ± SD (**p = 0.0013) Mann–Whitney. **F** Kaplan–Meier survival graph of mice with orthotopic xenografts of G7 GSC iRFP vector^CRISPR^ *n* = 9 (median survival 87 days) vs. MCL-1^CRISPR^ tumours *n* = 10 (median survival not definable, nd) post tumour cell implantation (****p < 0.0001) Log-rank (Mantel–Cox) test.

While it has previously been shown that GBM tumoursphere formation is promoted by high BCL-xL expression [44], little is known about the role of MCL-1 on GBM growth and maintenance. To explore the importance of MCL-1 in GBM formation and growth in vivo, we selected a tumourigenic cell line (G7 GSC) and deleted *MCL-1* using CRISPR/Cas9 genome editing. Western blot analysis confirmed efficient *MCL-1* deletion, while the expression of BCL-xL, SOX2 and GFAP was increased in the MCL-1^CRISPR^ cells (Supplementary Fig. 2A). MCL-1^CRISPR^ GSC were found to proliferate at the same rate as their vector^CRISPR^ counterparts and retained a capability to form neurospheres (Fig. 2A–C). We next investigated whether MCL-1 was required for tumourigenesis in vivo. iRFP-labelled vector^CRISPR^ and MCL-1^CRISPR^ G7 GSC were orthotopically injected in CD-1 nude mice and tumour growth was monitored with cranial magnetic resonance imaging (MRI) and iRFP signal detection [45]. We observed a substantial impairment of tumour growth in *MCL-1* deleted tumours (Fig. 2D, E; Supplementary Fig. 2B) that was reflected in the significantly prolonged survival of these mice (Fig. 2F). Importantly, IHC analysis of the end-stage tumours revealed an outgrowth of MCL-1 proficient tumour cells in the MCL-1^CRISPR^ xenografts with comparable Ki67 expression and almost absent cleaved Caspase 3 (Supplementary Fig. 2C–E). Outgrowth of MCL-1 proficient cells is likely due to incomplete deletion of MCL-1, underscoring the importance of MCL-1 in tumour growth. These data reveal a key role for MCL-1 in initiation and growth of GBM in vivo and identify MCL-1 as a promising therapeutic target. Our results also support an important pro-survival role for anti-apoptotic MCL-1 in GBM.

### GSC display increased apoptotic sensitivity and can be effectively killed by dual BCL-xL, MCL-1 inhibition

**Fig. 3:**
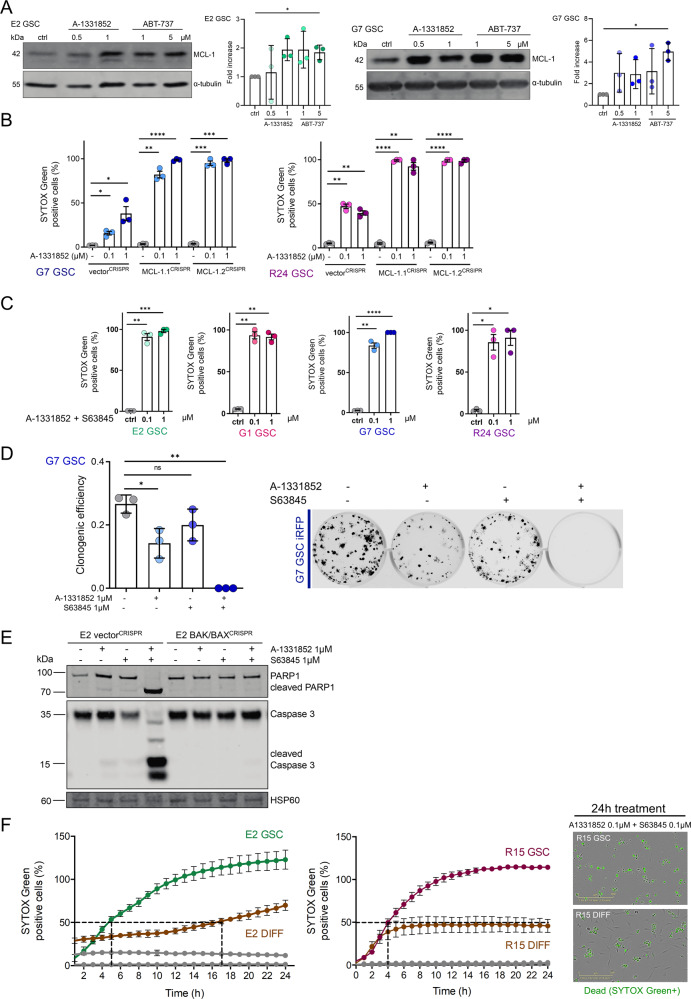
GSC display increased apoptotic priming and can be effectively killed by dual BCL-xL, MCL-1 inhibition. **A** G7 and E2 GSC were treated with DMSO (ctrl), A-1331852 or ABT-737 as indicated for 16 or 24 h, respectively, harvested and protein expression was analysed by immunoblot. α-tubulin served as loading control. Representative image from *n* = 3 independent experiments shown. Quantification of *n* = 3 independent experiments, error bars represent mean ± SD (*p ≤ 0.0292) Welch’s test. **B** G7 or R24 GSC vector^CRISPR^ vs. MCL-1.1^CRISPR^ and MCL-1.2^CRISPR^ were treated with A-1331852 for 24 h and analysed for cell viability using an IncuCyte imager and SYTOX Green exclusion. Percentage cell death was calculated by normalising against maximal cell death verified by visual inspection. Error bars represent mean ± SEM from *n* = 3 independent experiments (*p ≤ 0.0449, **p ≤ 0.004, ***p ≤ 0.0006, ****p < 0.0001) Welch’s test. **C** E2, G1, G7, R24 GSC were treated with a combination of A-1331852 and S63845 in indicated concentrations for 24 h and analysed for cell viability using an IncuCyte imager and SYTOX Green exclusion. Percentage cell death was calculated by normalising against maximal cell death verified by visual inspection. Error bars represent mean ± SEM from *n* = 3 independent experiments (*p ≤ 0.0123, **p ≤ 0.0022, ***p ≤ 0.0004, ****p < 0.0001) Welch’s test. **D** Clonogenic survival assay of G7 GSC iRFP treated with indicated drugs 16 h after plating 250 cells per well. Colonies counted manually after 14 days. Error bars represent mean ± SD from *n* = 3 independent experiments (ns *p* = 0.1355, *p = 0.0243, **p = 0.0038) Welch’s test. Representative images of a replicate in one independent repeat scanned on LICOR imager. **E** E2 GSC vector^CRISPR^ and BAK/BAX^CRISPR^ were treated as indicated for 2 h, harvested and protein expression was analysed by immunoblot. HSP60 served as loading control. Representative image from *n* = 3 independent experiments. **F** E2 and R15 GSC and paired DIFF cells were treated either with DMSO (grey) or a combination of A-1331852 and S63845 (both 0.1 μM) for 24 h and analysed for cell viability using an IncuCyte imager and SYTOX Green exclusion. Error bars represent mean ± SEM from *n* = 3 independent experiments. Representative IncuCyte images 24 h after treatment are shown.

Currently, no treatment regimen is able to achieve long-time remission of GBM, with tumours inevitably developing resistance to treatment and recurring, eventually leading to patient death [46]. Anti-apoptotic BCL-2 family members have overlapping binding affinities for several pro-apoptotic BH3-only proteins [8]. We asked whether GBM might circumvent single inhibitor treatment by compensatory upregulation of untargeted anti-apoptotic proteins. Indeed, upon treatment with A-1331852 or ABT-737 we found that levels of MCL-1 protein were increased in surviving GSC (Fig. 3A). We reasoned that upon displacement of pro-apoptotic proteins bound by BCL-xL via A-1331852, those might in turn bind to MCL-1, resulting in protein stabilisation and simultaneous MCL-1 overexpression. To test this hypothesis, we treated MCL-1^CRISPR^ G7 and R24 GSC with the BCL-xL inhibitors A-1331852 or ABT-263. Cell viability was measured by live-cell IncuCyte imaging with SYTOX Green exclusion or in a clonogenic survival assay. In all cases, *MCL-1* deletion significantly increased cellular sensitivity to the BCL-xL specific antagonist and navitoclax (Fig. 3B, Supplementary Fig. 3A). Similarly, dual inhibition of BCL-xL and MCL-1 with A-1331852 and S63845 displayed a synergistic treatment effect resulting in up to 100% cell death across a range of GSC, determined in both short term cell viability assays and long-term clonogenic survival assays (Fig. 3C, D, Supplementary Fig. 3B, C). This effect was observed even at 10-fold decreased doses compared to effective single treatment. Verifying on-target engagement of mitochondrial apoptosis, combined MCL-1 and BCL-xL inhibition led to Caspase 3 and PARP-1 cleavage as well as cell death in a BAK, BAX and caspase-dependent manner (Fig. 3E, Supplementary Fig. 3D–F).

Sensitivity of GBM cells to chemotherapy and ionising radiation inversely correlates with tumour cell stemness [6, 47, 48]. We therefore hypothesised that the differentiated counterparts (DIFF) of the patient-derived GBM stem-like cells may be more sensitive towards BH3-mimetic treatment. To ensure comparable culture conditions for GSC and DIFF, we conducted these experiments using 1% FCS containing Ad-DMEM medium during the experimental procedure. Cells were treated with A-1331852 and S63845 to inhibit BCL-xL and MCL-1 respectively and cell viability measured by live cell IncuCyte imaging and SYTOX Green exclusion. Following treatment with single BH3-mimetic, we found that cell viability was largely comparable for DIFF and GSC (Supplementary Fig. 3G). Surprisingly, following dual MCL-1 and BCL-xL inhibition, E2 and R15 GSC were more sensitive than DIFF cells; while >50% cell death was observed within about 5 h in GSC, it was not observed in DIFF cells until 16 h (Fig. 3F). Moreover, 100% cell death was not achieved in either of the DIFF cell lines. Together, these data suggest that GSCs are more primed for apoptotic cell death than DIFF cells. To investigate this further, we compared expression of pro- and anti-apoptotic BCL-2 proteins in the paired cell lines. Although more sensitive to apoptosis, GSCs displayed higher levels of anti-apoptotic BCL-2 proteins, BCL2, BCL-xL and MCL-1 than their differentiated counterparts (Supplementary Fig. 3H). In summary, these data indicate that GSC can display increased apoptotic sensitivity and reveal potent cytotoxic effects of dual-targeting BCL-xL and MCL-1.

### TrkB signalling regulates sensitivity of GSC to anti-apoptotic treatment

**Fig. 4:**
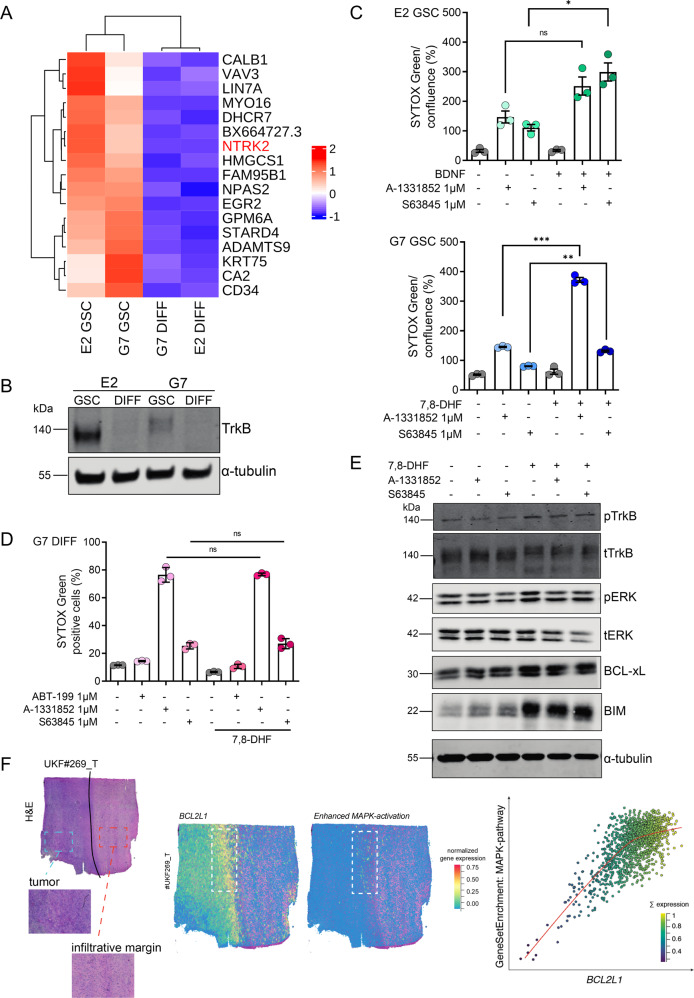
TrkB signalling regulates sensitivity of GSC to anti-apoptotic treatment. **A** Most differentially expressed genes in RNA sequencing analysis of E2 and G7 GSC vs. E2 and G7 DIFF. *NTRK2* codes for TrkB. **B** Immunoblot of TrkB in E2 and G7 GSC compared with paired DIFF cells. α-tubulin served as loading control. Representative image from *n* = 2 independent experiments. **C** E2 and G7 and GSC treated with BDNF (100 ng/mL) or 7,8-DHF (20 μg/mL) ± A-1331852 1 μM and S63845 1 μM for 24 h (after a 24-hour starvation period in 1% glutamine containing DMEM/F12) and analysed for cell viability using an IncuCyte imager and SYTOX Green exclusion. Error bars represent mean ± SEM from one of *n* = 3 independent experiments (E2 ns *p* = 0.0551, *p = 0.0166, G7 **p = 0.0013, ***p = 0.008) Welch’s test. **D** G7 DIFF treated as described in Fig. 4C and analysed for cell viability using an IncuCyte imager and SYTOX Green exclusion. Error bars represent mean + /-SEM from *n* = 3 independent experiments (ns, *p* ≥ 0.5662). **E** G7 GSC were treated with 7,8-DHF (20 μg/mL) ± A-1331852 1 μM and S63845 1 μM for 1 h, harvested and protein expression was analysed by immunoblot. α-tubulin served as loading control. Representative image from *n* = 3 independent experiments. **F** H&E of representative human GBM specimen (#UKF269_T) with predicted pattern visualised by the z-scored gene expression of *BCL2L1* and enhanced MAPK-pathway activation (middle) including analysis of their correlation (right).

We next sought to explore the differential apoptotic sensitivity of GSC and their isogenic differentiated counterparts. To this end, bulk RNA sequencing data from E2, G7 GSC and their DIFF counterparts was reanalysed [37]. Consistent with enrichment of GSC, RNAseq analysis revealed increased levels of CD34, a surface glycoprotein, first described as marker for haematopoietic progenitor cells [49]. Interestingly, high expression of *NTRK2* mRNA was detected in both GSC (Fig. 4A). This finding was validated in E2 and G7 GSC as well as R15 and R24 GSC via immunoblotting (Fig. 4B, Supplementary Fig. 4A). *NTRK2* the gene coding for the tropomycin receptor kinase B (TrkB) is primarily known for its function in neurodevelopment inducing downstream signalling upon binding of brain-derived neurotrophic factor (BDNF) [50]. Recently, Wang and colleagues have reported a role for TrkB-expressing cancer stem cells in GBM progression in response to BDNF stimulation by differentiated tumour cells [51]. As TrkB-mediated activation of MAPK and PI3K-AKT signalling is generally associated with cell survival [52], we hypothesised that BDNF-mediated TrkB stimulation might enable GSC to evade cell death. Unexpectedly, following stimulation of GSC with BDNF or 7,8-dihydroxyflavone (7,8-DHF), a specific TrkB agonist, we found that GSC were further sensitised to cell death following treatment with BH3-mimetics targeting BCL-xL and or MCL-1 (Fig. 4C, Supplementary Fig. 4B). This sensitising effect was not observed in the DIFF cells (Fig. 4D). DIFF cells express significantly lower levels of the TrkB receptor and therefore prove to be comparably unresponsive to BDNF stimulation (Supplementary Fig. 4C). BDNF-induced TrkB phosphorylation also led to increased BCL-xL protein expression, alongside stabilisation of the BIM protein downstream of MAPK signalling, independently of BCL-xL and MCL-1 inhibition (Fig. 4E). Matching to our in vitro findings, analysis of the GBM spatial transcriptomics data revealed a positive correlation between the expression of the BCL-xL and the activation of the MAPK-pathway within patient tumour samples (Fig. 4F). These data demonstrate a key role for BDNF-TrkB signalling in modulating the increased apoptotic sensitivity of GSCs.

### Combined BCL-xL and MCL-1 inhibition causes apoptosis in human GBM ex vivo

**Fig. 5:**
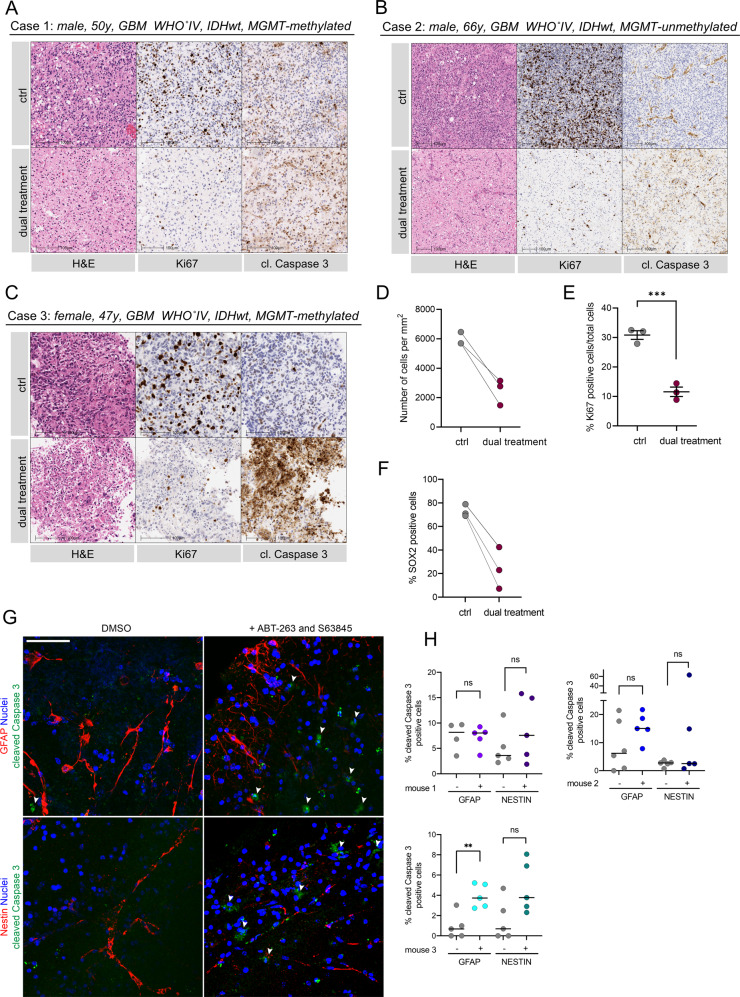
Combined BCL-xL and MCL-1 inhibition causes apoptosis in human GBM ex vivo. **A**–**C** Representative images of H&E, Ki67 and cleaved Caspase 3 IHC of three patients diagnosed with *IDHwt* GBM (case 1–3; ctrl = DMSO, dual treatment = A-1331852 1 μM + S63845 1 μM) (**D–F**) Quantification of cellularity, percentage Ki67 and SOX2 positive cells/total cell count in all three cases treated with the drugs described in (**A**–**C**) for 72 h. Error bars represent mean ± SEM (***p = 0.0009) Welch’s test. **G** Representative images of IF staining of GFAP, NESTIN (both red) and cleaved Caspase 3 (green) in the subventricular zone (SVZ) of mouse brain slices, cultured and treated with DMSO or a combination of ABT-263 5 μM and S63845 2 μM for 24 h, counterstaining with DAPI (blue). **H** Quantification of cleaved Caspase 3 positive cells normalised to nuclear count in single mice. Single dots represent analysed images. Error bars represent median (mouse 1: ns *p* = 0.9198, *p* = 0.3086; mouse 2: ns *p* = 0.149, *p* = 0.2974; mouse 3: ns *p* = 0.0593, **p = 0.0044) Welch’s test. Scale bars = 50 µm.

Current in vitro methodologies fail to recapitulate important aspects of the brain microenvironment and tissue context. Given this, we sought to use a more physiologically relevant model to investigate functional responses to BCL-xL and MCL-1 inhibition in GBM. For this purpose, we developed an assay tailored to the use of freshly resected human GBM to be cultured ex vivo as tissue slices that could be readily exposed to candidate drugs (experimental setup illustrated in Supplementary Fig. 5A). All three patients included in the study were diagnosed with *IDHwt* GBM according to the 2016 WHO classification [53]. Tissue slices were treated for 72 h in total. In all cases, we found that a combined therapy with A-1331852 and S63845 (BCL-xL and MCL-1 inhibition) significantly reduced tumour cell count compared with single drug treatment or control (Fig. 5A–D, Supplementary Fig. 5B–E). Moreover, dual treatment induced a significant reduction of cell proliferation (Ki67 IHC) and amount of SOX2 positive tumour cells (Fig. 5E, F, Supplementary Fig. 5F, G), while Caspase 3 cleavage was increased. This data indicates that the dual treatment efficiently targets GBM stem-like cells ex vivo. Importantly, the integrity of the brain tissue and vasculature was maintained (Supplementary Fig. 5H).

In recent years, selective MCL-1 and BCL-xL inhibitors have been developed that show effective in vivo potency [38, 39]. However, systemic exposure to both inhibitors is limited due to its combined toxicity [54]. Because the blood-brain-barrier is only permissible to certain drugs, local or intrathecal drug application [55] might allow to circumvent systemic side effects in a clinical setting. To explore potential toxicities to resident brain cells, we used human neural stem cells (hNS) and astrocytes (hAS) and treated them in vitro. Here, we found that hNS were insensitive to BH3-mimetics (Supplementary Fig. 5I). In case of cultured hAS, we found that they were highly sensitive towards BH3-mimetic treatments (Supplementary Fig. 5J). However, the in vivo relevance of this sensitivity is unclear since proliferative astrocytes were cultured in serum-containing medium to allow for differentiation and propagation. Addition of serum has been shown to induce irreversible reactive changes not seen in quiescent astrocytes directly obtained from brains [56, 57]. For those reasons, we used the mouse brain slice toxicity assay as it allowed to investigate all prevalent cell types with their normal phenotype and within their physiological environment. We deployed brain slices from 11-week old adult mice and exposed them to dual treatment with the indicated BH3-mimetics. In regions of the subventricular zone, an important neural stem cell niche, we detected only a moderate increase of cleaved Caspase 3 in a fraction of glial and neural progenitor cells (GFAP and NESTIN IF stain) (Fig. 5G, H). In summary, these data suggest that dual targeting of BCL-xL and MCL-1 may provide a novel therapeutic approach to treat GBM.

### Sequential BCL-xL and MCL-1 inhibition effectively kills GSC and promotes tumour regression in vivo

**Fig. 6:**
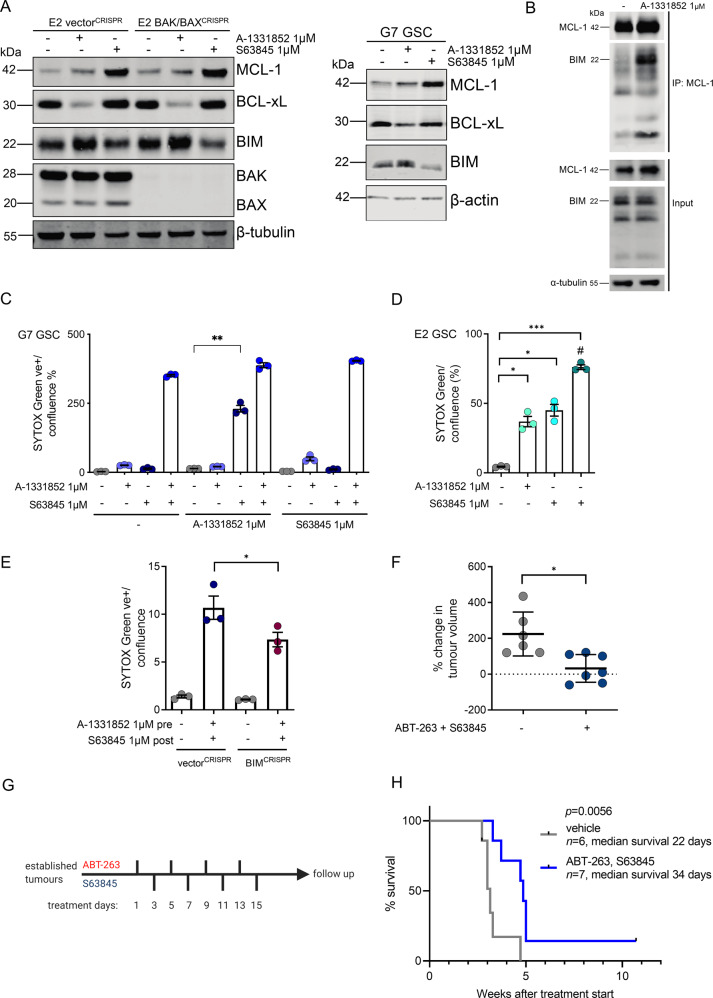
Priming with BCL-xL inhibition renders GSC vulnerable to MCL-1 inhibition, promoting tumour regression in vivo. **A** E2 GSC vector^CRISPR^ and BAK/BAX^CRISPR^, G7 GSC were treated with DMSO (-), A-1331852 and S63845 as indicated for 24 h, harvested and protein expression was analysed by immunoblot. E2: representative image from *n* = 3 independent experiments. β-tubulin served as loading control. G7: representative image from *n* = 1 experiment. β-actin served as loading control. **B** Direct binding interactions between MCL-1 and BIM were immunoprecipitated and interacting proteins were detected by western blot in G7 GSC treated for 16 h with A-1331852 (Input total cell lysate, IP immunoprecipitated fraction). Representative image from *n* = 2 independent experiments. **C** G7 GSC were treated with DMSO, A-1331852 or S63852 1 μM for 48 h, followed by 24 h drug washout with exchange to fresh medium and treatment with indicated drugs for 24 h. For cell viability analysis, IncuCyte imager and SYTOX Green exclusion was used. Error bars represent mean ± SEM from *n* = 3 independent experiments (**p = 0.003) Welch’s test. **D** E2 GSC were pre-treated with DMSO or A-1331852 1 µM (#) for 24 h, followed by 24 h drug washout with exchange to fresh medium and treatment with indicated drugs for 48 h. For cell viability analysis, IncuCyte imager and SYTOX Green exclusion was used. Error bars represent mean ± SEM from *n* = 3 independent experiments (**p ≤ 0.0123, ***p = 0.0004) Welch’s test. **E** G7 GSC vector^CRISPR^ and BIM^CRISPR^ were treated for 48 h with A-1331852 1 µM, followed by 24 h drug washout with exchange to fresh medium and treatment with S63845 1 µM for 24 h. For cell viability analysis, IncuCyte imager and SYTOX Green exclusion was used. Error bars represent mean ± SEM from *n* = 3 independent experiments (*p = 0.0132) Welch’s test. **F** Percent U87MG subcutaneous tumour volume change at the end of 2 weeks alternating treatment with ABT-263, followed by S63845, relative to tumour size at start. Treatment commenced when tumours were >5 mm diameter. *n* = 6 vehicle treated (grey dots) and *n* = 7 drug treated (blue dots). Bars are mean ± SD (*p = 0.0104) Welch’s test. **G** Schematic of treatment schedule for in vivo study. Upon tumour establishment ABT-263 (20 mg/kg) was administered via oral gavage, 48 h later followed by S63845 (25 mg/kg) via tail vein injection. The treatment was given over a period of 15 days. **H** Kaplan–Meier survival analysis of U87 vehicle treated (grey line, *n* = 6, median survival 22 days) vs. U87 drug treated (blue line, *n* = 7, median survival 34 days) since treatment start (**p = 0.0056) Log-rank (Mantel–Cox) test.

Given the potency of joint BCL-xL/MCL-1 inhibition in our in vitro and ex vivo findings, we sought to maximise this combinatorial effect whilst mitigating possible systemic toxicity. To address this, we investigated which pro-apoptotic proteins are involved in regulating intrinsic apoptosis in GBM. Upon single agent treatment with A-1331852, we observed upregulation of the BH3-only protein BIM as well as anti-apoptotic MCL-1. This was seen in both control and BAX/BAK deficient GSC (Fig. 6A, Supplementary Fig. 6A). BIM is an important BH3-only protein in the canonical apoptotic pathway where it functions by regulating both BCL-xL and MCL-1 mediated cell death responses [58]. We hypothesised that MCL-1 might bind and neutralise BIM that is released by the A-1331852 complexing to BCL-xL. Accordingly, immunoprecipitation of MCL-1 following treatment with A-1331852 revealed increased binding of BIM to MCL-1 (Fig. 6B). To explore whether this mechanism could be therapeutically exploited, we questioned whether BCL-xL inhibition would render GSC more sensitive to subsequent MCL-1 inhibition. GSC were treated with a BH3-mimetic targeting either BCL-xL or MCL-1 for up to 48 h followed by a washout and 24 h treatment pause. Subsequently, the complementary inhibitor was applied for up to 48 h. Whereas prior inhibition with MCL-1 inhibitor failed to sensitise the cells to BCL-xL inhibition, pre-treatment with the BCL-xL inhibitor substantially increased the susceptibility of GSC to subsequent MCL-1 inhibition (Fig. 6C, D). To further investigate the relevance of BIM in mediating apoptosis following BCL-xL and MCL-1 inhibition, we deleted BIM by CRISPR/Cas9 genome editing (Supplementary Fig. 6A). Using IncuCyte live cell imaging and SYTOX Green exclusion to detect cell death under treatment we observed that knockout of BIM did not impede the sensitivity of G7 GSC to concurrent dual BCL-xL and MCL-1 inhibition (Supplementary Fig. 6B). However, after pre-treatment with BCL-xL inhibitor A-1331852 G7 BIM^CRISPR^ GSC were less primed for following MCL-1 inhibition compared to their vector^CRISPR^ counterparts (Fig. 6E). These results indicate that Bcl-xL inhibition mediates sensitisation of GSC to subsequent MCL-1 neutralisation via pro-apoptotic BIM. Finally, we aimed to investigate the potency of alternating BH3-mimetic treatments in GBM in vivo. Due to the poor blood-brain-barrier penetrance of ABT-263, we chose a subcutaneous model. As the GSC used in our study do not grow as subcutaneous xenografts we explored whether human U87MG respond to combined and alternating inhibition of BCL-xL and MCL-1 in like manner to GSC. Indeed, we found that dual inhibition of BCL-xL and MCL-1 induced substantial cell death in U87MG neurospheres (Supplementary Fig. 6C). Testing different treatment regimens using a clonogenic survival assay, we found a profound decrease in colony formation upon alternating treatment with ABT-263 and S63845 (Supplementary Fig. 6D). In our in vivo cohort, mice were treated with vehicle or alternating therapy with ABT-263 and S63845 every 48 h for two weeks upon tumour establishment (treatment schematic illustrated in Fig. 6G). Compared to mice receiving vehicle control, mice treated with the sequential therapy showed significant attenuation and/or regression of tumours (Fig. 6F). Most importantly, we observed significantly improved survival in mice treated with ABT-263, followed by S63845 (Fig. 6H). One mouse had a complete tumour regression after sequential treatment with no reoccurrence of the subcutaneous tumour over an 8-week follow up period. With this treatment schedule no significant weight loss (Supplementary Fig. 6E) or signs of neurological deficits were detected in mice. Histopathological analysis of tumours reaching clinical end point showed a higher prevalence of a large central necrotic areas in within the treatment cohort compared to vehicle control (83% vs. 33%) (Supplementary Fig. 6F). Collectively, these data demonstrate the therapeutic potential of sequential BCL-xL and MCL-1 inhibition in GBM.

## Discussion

Largely due to a dearth of effective treatment options, GBM patients have a dismal prognosis [46]. Analysis of the TCGA glioma dataset reveals even a worsened survival rate for patients with high *BCL-xL* and *MCL-1* gene expression. Addressing this, we investigated the therapeutic potential of targeting anti-apoptotic BCL-2 proteins in GBM. Our analysis showed high expression of anti-apoptotic BCL-xL and MCL-1 in GBM. Moreover, we also observed increased expression of BCL-xL and MCL-1 in GBM stem-like cells - a population of cells that are key for GBM development and treatment resistance in vivo [5, 6]. Rather than promoting apoptotic resistance, elevated anti-apoptotic BCL-xL and MCL-1 expression in GSC compared to isogenic DIFF correlated with increased susceptibility to targeted inhibition using BH3-mimetics. This indicates that GSC are inherently primed for apoptosis. Exploiting this, we found that GBM were sensitive to BH3-mimetics targeting either MCL-1 or BCL-xL. Crucially, alternating dosing with BCL-xL followed by MCL-1 specific BH3-mimetics, led to durable treatment responses with preceding BCL-xL inhibition sensitising to MCL-1 inhibition in vivo. These data highlight the therapeutic potential of targeting BCL-xL and MCL-1 in GBM.

Recently, highly specific and potent BH3-mimetics have been developed to target BCL-2, BCL-xL or MCL-1 [14, 18, 38, 39]. We used these to probe the individual dependencies of GBM in a panel of patient-derived GSC. Importantly, we found that GBM cells are largely dependent on BCL-xL or MCL-1 for survival, whereas BCL-2 plays a lesser role. Genetic deletion of *MCL-1* corroborated its key role in both promotion and maintenance of GBM. Consistent with our findings, indirect targeting of MCL-1 through CDK7 inhibition, causing transcriptional repression, sensitises GBM cell lines to ABT-263 [59]. Further, we could demonstrate that both BCL-xL and MCL-1 are highly expressed, not only in GBM tumour cores, but also in GBM stem-like cells compared to their isogenic differentiated counterparts and astrocytes. The high expression and dependence of GBM on anti-apoptotic BCL-2 function is consistent with an increased state of apoptotic priming. As Sarosiek and colleagues have demonstrated, the tissue of origin plays a major role in determining the apoptotic sensitivity of a cell [35], therefore the high dependency of stem-like cells on BCL-xL and MCL-1 might relate to their resemblance of cerebral precursor cells. Both anti-apoptotic proteins play a major role in neurogenesis, while with brain maturation neurons become refractory to apoptotic cell death [33, 35].

Unexpectedly, we observed that in comparison to their differentiated counterparts, stem-like cells were more susceptible to BH3-mimetic treatment. Investigating the basis of the differential apoptotic priming we identified NTRK2, a stem-cell specific surface receptor [51] as a key component. NTRK2 signalling, mediated by the soluble brain-derived neurotrophic factor BDNF, plays a major role in cell survival promotion of growth in glial tumours [51, 60]. Surprisingly, we found that BDNF stimulation led to increased apoptotic sensitivity. This result reinforces the notion that stem-like cells are especially dependent on the tight regulation of apoptotic sensitivity (Supplementary Fig. 6G). More profound understanding of the tumour-environmental context should shed light on how these interactions can be therapeutically exploited to maximise treatment efficacy of BH3-mimetics.

To facilitate translation to the clinic, we developed an ex vivo assay to investigate chemosensitivity of fresh patient-derived GBM tissue to BCL-2 targeting BH3-mimetics. Across different freshly resected *IDHwt* GBM samples, we found that targeting both MCL-1 and BCL-xL led to an extensive induction of apoptosis and sustained reduction in tumour cell viability ex vivo, without compromising tissue and vessel integrity. Following inhibition of BCL-xL we found an increased abundance of BIM bound to MCL-1, leading to a sensitisation of the GBM cells to MCL-1 antagonists. This mirrors studies in haematologic malignancies where the susceptibility to BH3-mimetics was dependent on BCL-2 complexed to BIM [61, 62] and increased BIM levels sensitised to BCL-2 inhibition [63].

To circumvent reported systemic toxicity, we developed a sequential treatment schedule. Due to the limited drug permeability of the blood-brain-barrier we resorted to test this treatment schedule using the GBM cell line U87MG in a subcutaneous xenograft model. The effect of sequential treatment observed in vitro could also be recapitulated in vivo with a profound regression of tumour size and significant survival benefit. This contrasts with previous findings showing lack of effect of single BH3-mimetic treatment in U87 tumours in vivo, thus emphasising a key role for multiple BCL-2 proteins in enabling GBM survival [27]. Importantly, the brain, due to its blood-brain-barrier, provides unique opportunities for drug delivery. For instance, local drug delivery or intrathecal chemotherapy can be exerted to use the blood-brain-barrier and in turn circumvent systemic side effects [55]. In summary, these data provide a rationale for further investigating alternating inhibition of BCL-xL and MCL-1 pro-survival function in GBM to maximise the therapeutic effect.

## Materials and methods

### GBM cell lines and cell culture reagents

Patient-derived GBM stem-like cells (E2, G1, G7, R9, R15, R24 GSC), obtained from surgical resection specimens of anonymised patients as described [64, 65], were kindly provided by Prof. Colin Watts. Human astrocytes (hAS) and neural stem cells (hNS) were originally provided by Prof. Steven Pollard as human foetal neural stem cells [36, 66]. U87MG were given by Prof. Anthony Chalmers. GSC and U87MG were cultured in serum-free Advanced Dulbecco’s modified Eagle’s medium F12 (Thermo Fisher Scientific), supplemented with 2 mM glutamine, 4 μg/ml heparin (Sigma), 1% B27 (Thermo Fisher Scientific), 0.5% N2 (Thermo Fisher Scientific), 20 ng/ml EGF and 10 ng/ml FGF-2 (Thermo Fisher Scientific). DIFF cells were cultured in 10% foetal calf serum (FCS) containing high-glucose DMEM (Thermo Fisher Scientific) complemented with 2 mM glutamine. All cells were kept in 37 °C incubator at 5% CO_2_ and, when grown as monolayers on Matrigel (Corning) pre-coated plates or as spheres in uncoated plates. Cell expansion of hNS was carried out in serum-free medium supplemented with N2, B27, EGF, 20 ng/ml FGF-2 and 1 mg/ml Laminin (Sigma) [66]. hAS were differentiated by seven days culture in serum containing medium as described before [67]. For all experiments, cells were used up to ten passages after thawing. All cell lines used were routinely tested for mycoplasma.

For our in vitro studies the following drugs and chemicals were used: ABT-199 (AdooQ BioScience, #A12500-50), ABT-263 (ApexBio, #A3007), ABT-737 (ApexBio, #A8193), A1331852 and A1155463 (ApexBio, #B6164 and #B6163), S63845 (Chemgood, #C-1370), Actinomycin D (Calbiochem, #114666), Temozolomide (Sigma, #T2577), q-VD-OPh (QVD, AdooQ BioScience, #A14915-25), SYTOX Green (Thermo Fisher Scientific, #S7020), Brain-derived neurotrophic factor (BDNF; Peprotech, #450-02), 7,8-Dihydroxyflavone hydrate (7,8-DHF; Merck, #D5446).

### Lentiviral transduction

GSC transduction was performed using CRISPR/Cas9 genome editing with the following guide sequences:

*hBAX: 5*′*-AGTAGAAAAGGGCGACAACC-3*′

*hBAK: 5*′*-GCCATGCTGGTAGACGTGTA-3*′

*hMCL-1.1: 5*’*-GGGTAGTGACCCGTCCGTAC-3*’

*hMCL-1.2: 5*’*-GTATCACAGACGTTCTCGTA-3*’

*hBIM: 5*’*- TACCCATTGCACTGAGATAG-3*’

For stable cell line generation HEK293-FT cells (4 × 10^6^ in a 10 cm dish) were transfected using 4 μg polyethylenimine (PEI, Polysciences) per μg plasmid DNA with the LentiCRISPRv2-puro (Addgene #52961) or LentiCRISPRv2-blasti [68] backbone, lentiviral transfer vector plasmid, packaging plasmid (Addgene #14887) and envelope plasmid pUVSVG (Addgene #8454), mixed in a 4:2:1 ratio. DNA/PEI mixtures were incubated at room temperature for 10–15 min, prior to application on HEK293-FTs. 24 and 48 h later, virus containing supernatant was harvested and filtered (0.45 μM). Virus was extracted using Lenti-X concentrator (Clontech Takara) according to the manufacturer’s instructions. The virus containing pellet was resuspended in serum-free stem-cell medium and target cells were infected in the presence of 1 μg/ml polybrene (Sigma Aldrich). Two days following infection, cells were selected by growth in puromycin (E2: 1 μg/ml, G7: 0.5 μg/ml; Sigma Aldrich) or blasticidin (G7, R15 and R24: 10 μg/ml; InvivoGen) containing medium. As described previously, plasmids encoding iRFP IRES puro have been inserted into a pBABE vector [45].

### Cell proliferation and live cell viability assay

Cell death and cell confluence were determined using live cell imaging in the IncuCyte Zoom and S3 (Sartorius). For cell confluence 50 × 10^3^ cells were seeded in Matrigel-coated 6-well plates. Cell area per well was measured using IncuCyte imaging analysis software (Sartorius). For cell death assays, 6 × 10^3^ or 12 × 10^3^ GSC, 10 × 10^3^ hAS and 1 × 10^5^ hNS per well were seeded in Matrigel-coated 96-well plates and treated with the indicated drugs in the presence of 30 nM SYTOX Green. Plates were applied to the IncuCyte imager at 37 °C in a humidified 95% air/ 5% CO_2_ incubator. Every hour, two images per well were taken over a period of 24–48 h. Images were presented in green phase contrast at 10x magnification. For image quantification IncuCyte imaging analysis software was used. Percentage cell death was calculated by normalising against maximal cell death control upon 24–48 h treatment (1 µM Actinomycin D, 10 µM ABT-737 and 1 µM S63845). Alternatively, 100% cell death control was verified by visual inspection of IncuCyte images, where 100% SYTOX Green positive cells = total cell count.

### Clonogenic survival assay and radiation

GSC were seeded at a density of 250 cells per well in Matrigel-coated, 6-well plates with three technical repeats per experiment and left to adhere overnight. After 16 h cells were treated as indicated for 24 h, followed by replacement of fresh media. For the drug-radiation treatment cells were treated with vehicle or ABT-737 in fresh media 24 h prior to irradiation in a regularly calibrated x-ray cabinet at room temperature with (195 kV, 15 mA, 0.5 mm Cu filter, X-Strahl).

Cells were left to form colonies for 2 to 3 weeks prior to methanol fixation and crystal violet staining. Visible colonies consisting of minimum 50 cells were counted manually.

### Neurosphere formation assay

G7 GSC vector^CRISPR^ and MCL-1^CRISPR^ were seeded at a density of 1, 5, 10, 20 and 50 cells per well in uncoated 96-well plates. Serum-free stem-cell medium was refreshed every week. Spheres were left to grow for 14 days before manual scoring of the 60 inner wells. Extreme limiting dilution analysis was performed using of the publicly available software https://bioinf.wehi.edu.au/software/elda [69].

### Immunoblotting, immunoprecipitation and antibodies

GSC were lysed and collected in RIPA buffer (50 mM Tris-HCl pH 7.5, 150 mM NaCl, 1 mM EDTA, 1% NP-40), supplemented with complete protease inhibitor (Roche) and PhosSTOP (Roche). Protein concentration was determined using Pierce BCA protein assay kit (Thermo Fisher Scientific) and protein lysates were subjected to electrophoresis through SDS–PAGE or 4-12% NuPage Bis-Tris protein gels (Thermo Fisher Scientific) followed by blotting onto nitrocellulose membranes. After blocking in 5% non-fat, dry milk or 2% BSA (Roche), membranes were probed with primary antibody (dilution 1:1000) BAK (Cell Signaling #12105), BAX (Cell Signaling #2772), BCL-2 (Cell Signaling #2762), BCL-xL (Cell Signaling #2762), MCL-1 (Cell Signaling #5453), BIM (Cell Signaling #2933), TrkB (Cell Signaling #4603), pTrkA (Tyr674/675)/pTrkB (Tyr706/707) (Cell Signaling #4621), ERK1/2 (Cell Signaling #4695), pERK1/2 (Cell Signaling #4370), AKT (Cell Signaling #9272), pAKT (Ser473; Cell Signaling #4066), Caspase 3 (Cell Signaling #9662), cleaved Caspase 3 (Cell Signaling #9664), PARP1 (Cell Signaling #9532) and SOX2 (Abcam #ab92494), NESTIN (Abcam #ab22035), GFAP (Santa Cruz #SC-6170) at 4 °C overnight in blocking buffer. α-tubulin (Sigma #T5168, 1:5000), β-tubulin (Cell Signaling #2146, 1:5000), HSP60 (Cell Signaling #4870, 1:1000), or actin (Sigma #A4700, 1:5000) served as loading controls. Each blot was probed with primary antibodies and a loading control. Representative loading controls are shown in figures. Membranes were incubated with Li-Cor secondary antibodies (IRDye 680RD donkey anti-mouse, IRDye 800CW donkey anti-rabbit, IRDye 800CW donkey anti-goat) for 1 h at room temperature. Scans of uncropped Western blots are shown in supplemental data.

For immunoprecipitation (IP), rabbit antibodies were coupled to magnetic beads conjugated to anti-rabbit IgG (Dynabeads Sheep anti-rabbit IgG, Invitrogen, #11203D). The buffer containing 200 mM NaCl, 75 mM Tris-HCl pH 7, 15 mM NaF, 1.5 mM Na3VO4, 7.5 mM EDTA, 7.5 mM EGTA, 0.15% (v/v) Tween-20 and protein inhibitors (Thermo Fisher) were used to prepare cell lysates. Lysates were passed several times through a 26-gauge needle followed by centrifugation at 10,000 g for 5 min at 4 °C. Lysates were added to the beads and rotated for 2 h at 4 °C. After washes in Tween-20-containing buffer, lysates were analysed by immunoblotting.

Blots were imaged using Li-Cor Odyssey CLx (Li-Cor), acquired and processed using Image-Studio software (Li-Cor) and subsequently arranged using Adobe Illustrator.

### Orthotopic intracranial and subcutaneous xenografts

All mouse experiments were carried out in accordance with the Animals Act 1986 (Scientific Procedures on living animals) and the regulatory guidelines of the EU Directive 2010 under project licences PPL P4A277133 and PP6345023 and ethical review (University of Glasgow). Sample sizes were based on our previous experiences with intracranial and subcutaneous GBM xenografts using the same cell lines.

For intracranial xenografts, 7-week old female CD1-nude mice (Charles River, UK) were orthotopically injected with 1 × 10^5^ iRFP-labelled vector^CRISPR^ and MCL-1^CRISPR^ G7 GSC into the right striatum. Mice were monitored for the duration of the experiment and humanely sacrificed when they showed neurological (hemiparesis, paraplegia) or general symptoms (hunched posture, reduced mobility, and/or weight loss >20%).

For subcutaneous xenografts, 1 × 10^6^ U87MG cells, previously cultured in stem-cell medium, were diluted in PBS and 50% growth factor reduced Matrigel and injected in the right flank of 8-week old female CD1-nude mice (Charles River, UK). When tumours reached ~5 mm in diameter treatment was commenced and mice were allocated into treatment arms to equate mean tumour volume in a non-randomised manner. For in vivo dosing, ABT-263 (ChemieTek #A263) was dissolved in 10% ethanol, 30% PEG glycol 400 and 60% Phosal 50 PG at 20 mg/kg and administered via oral gavage. S63845 (ChemieTek #S63845) was prepared in 2% vitamin E/d-α-tocopheryl polyethylene glycol 1000 succinate (Sigma) immediately prior to IV administration by tail vein injection at 25 mg/kg. Mice were treated with a 48-h pause between drug administrations over a 14 days period. Tumour growth was monitored by caliper measurement three times per week and volume calculated using the equation ([length x width^2^]/2). Upon completion of treatment mice were followed up to clinical end point (15 mm tumour diameter or ulceration of the tumour), at which they were euthanised.

### Intravital cranial iRFP imaging and magnetic resonance imaging (MRI)

To examine intravital, intracranial tumour growth in animals bearing iRFP-positive G7 GSC, mice were monitored by PEARL imaging (Li-Cor) as previously described [45]. MRI scans performed on brain tumour bearing mice using a nanoScan PET/MRI scanner (Mediso Medical Imaging Systems, Hungary). Mice were maintained under inhaled isoflurane anaesthesia (induction 5% v/v; maintenance 1.5–2.0% v/v) in the medical air during imaging procedure duration. Whole brain T2 Fast Spin Echo (FSE) 3D Axial Sequences (slice thickness 1.0 mm, repetition time (TR) 2000 msec, echo time (TE) 83.7 msec, Flip Angle 90 degrees) were used to acquire MRI scans. For assessments of scans, volume-of-interest (VOI) was manually drawn around the tumour region on MRI scans by visual inspection. Separate VOI were drawn for each scan to adjust for the position of the mice on the scanner and tumour size.

### Patient-derived GBM specimens and tissue culture

GBM specimens were obtained from surplus tumour tissue resected from patients treated within the OPARATIC study (NCT01390571). Patients had consented for use of surplus tissue for future research projects.

Fresh GBM tissues were obtained from surplus surgical resection tissue from patients at the Queen Elizabeth University Hospital (QEUH) in Glasgow upon written informed patient consent and after review by neuropathology in accordance with the NHS GG&C ethical committee review (Biorepository Application No. 432). The patient study was conducted in accordance with the Declaration of Helsinki. Neuropathological diagnosis and selected patient information are displayed in the figures. Further, details of these patients are restricted by institutional requirements. All experiments were performed conform to relevant regulatory standards of the CRUK Beatson Institute. Fresh samples were attenuated in 2% low gelling temperature agarose (Merck) and cut into 350 μm thick slices using the McIlwain tissue slicer (Campden Instruments). Tissue slices were dissected under the microscope in ice cold PBS before they were transferred on top of hydrophilic Millicell cell culture inserts (Merck Millipore) into serum-free Advanced Dulbecco’s modified Eagle’s medium F12 supplemented with 0.5% N2, 1% B27, 1% glutamine and 1% penicillin-streptomycin and left to equilibrate for 24 h at 37 °C in a humidified 95% air/ 5% CO_2_ incubator, before treatment with indicated drugs for 72 h. Following PBS washes brains were fixed in 4% paraformaldehyde (PFA) overnight.

### Organotypic adult mouse brain slice culture

Extracted brains from three female 11-week-old C57BL/6 J mice were transferred to sterile PBS on ice, divided into both hemispheres and cut into coronal, 100 μm thick slices using a vibratome (Campden Instruments 5100mz, advance speed 1 mm/sec, oscillation amplitude 1.5 mm, 80 Hz). Up to 5 slices per hemisphere were cut around the subventricular zone (SVZ). Slices were cultured on top of cell culture inserts in neurobasal medium as described in the previous section and left to equilibrate for 1 h at 37 °C in a humidified 95% air/ 5% CO_2_ incubator before treatment with indicated drugs for 24 h. After PBS washes slices were fixed in 4% PFA overnight.

### Immunohistochemistry (IHC) and immunofluorescence (IF)

H&E staining and IHC was performed on 4 μm formalin fixed paraffin embedded (FFPE) sections. For BCL-xL (Cell Signalling #2764), cleaved Caspase 3 (Cell Signalling #9661) and MCL-1 (Abcam #ab32087) IHC staining the Leica Bond Rx Autostainer was used. All FFPE sections underwent on-board antigen retrieval for 20 min using ER2 retrieval buffer (Leica, UK) before staining at a previously optimised dilution (BCL-xL 1:500; cleaved Caspase 3 1:500; MCL-1 1:200) and visualised with Liquid DAB (Agilent, UK). Ki67 Mib1 (Agilent #M7240) staining was performed on a Dako Autostainer Link 48 using high pH TRS retrieval buffer performed in a PT module (20 mins at 97 °C). Ki67 was applied at 1:100 dilution before visualising using Liquid DAB. For SOX2, IHC epitope retrieval was achieved by heating to 98 °C in pH6 citrate buffer for 60 min before proceeding as per the manufacturer’s instructions with SOX2 antibody used at a dilution of 1:500. Scanning and image analysis was conducted using Halo (Indica Labs). Algorithms were optimised for each stain individually and automated, quantitative analysis undertaken with Halo software (Indica Labs).

For IF staining tissue slices were permeabilised and blocked in PBS with 10% NGS, 1% BSA, 0.3% TX-100 and 0.05% Azide for 1 h at room temperature. After washes with 10% NGS, 1% BSA, 0.1% TX-100 and 0.05% Azide containing buffer slices were incubated with primary antibodies (NESTIN 1:300, GFAP 1:400, cl. Caspase 3 1:400) in washing buffer for 72 h at 4 °C. After washes slices were incubated in secondary antibodies (1:200, Alexa Fluor 568 goat anti-mouse (#A11004), Alexa Fluor 488 goat anti-rabbit (#A11034), Life Technologies) in washing buffer for 24 h. Following washes in PBS tissues were counterstained with DAPI (VECTASHIELD, LSBio) and mounted in gaskets (BioRad Seal Frame Incubation Chambers) on glass cover slips. Images were acquired using a Zeiss 710 laser scanning microscope with an EC Plan-Neofluar 40x/1.30 Oil DIC M27 objective and Zen 2.3 SP1 FP3 (black edition) software. 70 µm Z-stacks were acquired at 2.5 µm intervals and Maximum Intensity Projections (MIPs) were generated using Zen 2.1 (blue edition). Image processing was performed using Fiji (ImageJ 1.53c). Cleaved Caspase 3 positive cells were counted manually and nuclei were counted automatically using CellProfiler (Version 4.0.7).

### In silico and transcriptomic analysis

REMBRANDT microarray data was obtained from gliovis.bioinfo.cnio.es. Data was filtered for histology and tumour grading.

RNA sequencing data was obtained from a previously published GBM database [37]. In order to determine the most differentially expressed genes, calculation of expression rank product was employed to assess relative gene expression in paired GSC and DIFF cell lines [70]. Only results >10 reads were incorporated.

### Spatial transcriptomic analysis

Spatially resolved transcriptomic data from the “Freiburg Atlas of Spatial Biology” of the University Medical Center Freiburg were used. Sixteen de novo GBM of *IDH* wild type were included in the analysis. Data analysis was performed using SPATA2 (https://themilolab.github.io/SPATA2/) including the wrapper functions available in the SPATAwrappers package (https://github.com/heilandd/SPATAwrappers). Gene expression visualisation at spatial resolution was performed by the *plotSurface()* function. Overlap and spatially weighted correlation analysis was performed by the *SPATAwrappers::-inferSpatial.mc()* function. Statistical analysis was performed by one-way anova and paired *t* test statistics. P values were adjusted by the Benjamini–Hochberg method.

### Statistical analyses

Unless stated otherwise, two tailed, unpaired *t* test with Welch’s correction (Welch’s test) or Mann–Whitney test were used for comparison of two experimental groups. For tumour related Kaplan–Meier survival curves Mantel–Cox (Log-rank) was plotted. These statistical analyses were executed with Prism software version 9 (GraphPad, La Jolla, CA, USA).

## Supplementary information

Suppemental Figure 1

Suppemental Figure 2

Suppemental Figure 4

Suppemental Figure 3

Suppemental Figure 5

Suppemental Figure 6

Source data - uncropped blots

Supplemental Figure Legends

Change of authorship form

Author contribution list

Checklist

## Data Availability

The REMBRANDT microarray data used in this study is available in the GlioVis database under [gliovis.bioinfo.cnio.es]. Spatial transcriptomic RNA-sequencing data derived from the “Freiburg Atlas of Spatial Biology”. Data analysis was performed using SPATA2 (https://themilolab.github.io/SPATA2/) including the wrapper functions available in the SPATA wrappers package (https://github.com/heilandd/SPATAwrappers). Source data are provided as additional file with this paper. The remaining data are available within the main article and supplementary information. Further requests for resources, raw data and reagents should be directed to: stephen.tait@glasgow.ac.uk.
