# Supplementary figures and images for: Increased apoptotic sensitivity of glioblastoma enables therapeutic targeting by BH3-mimetics
Source: Cell Death Differ. 2022 Apr 26;29(10):2089–104. doi: 10.1038/s41418-022-01001-3 (PMC9525582; doi:10.1038/s41418-022-01001-3)

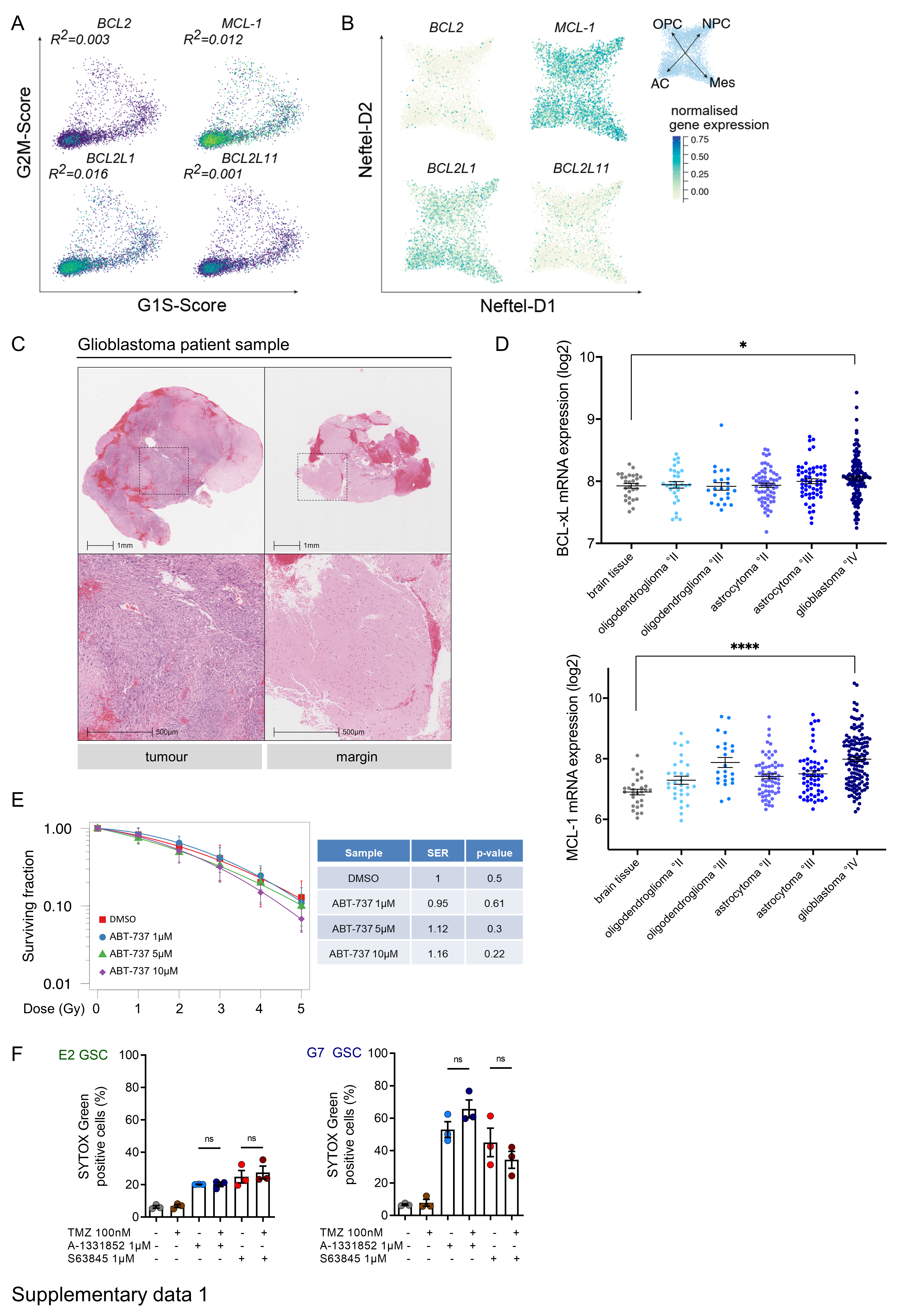

Supplement: Supplementary file 1 — Suppemental Figure 1 [file 41418_2022_1001_MOESM1_ESM.tif]

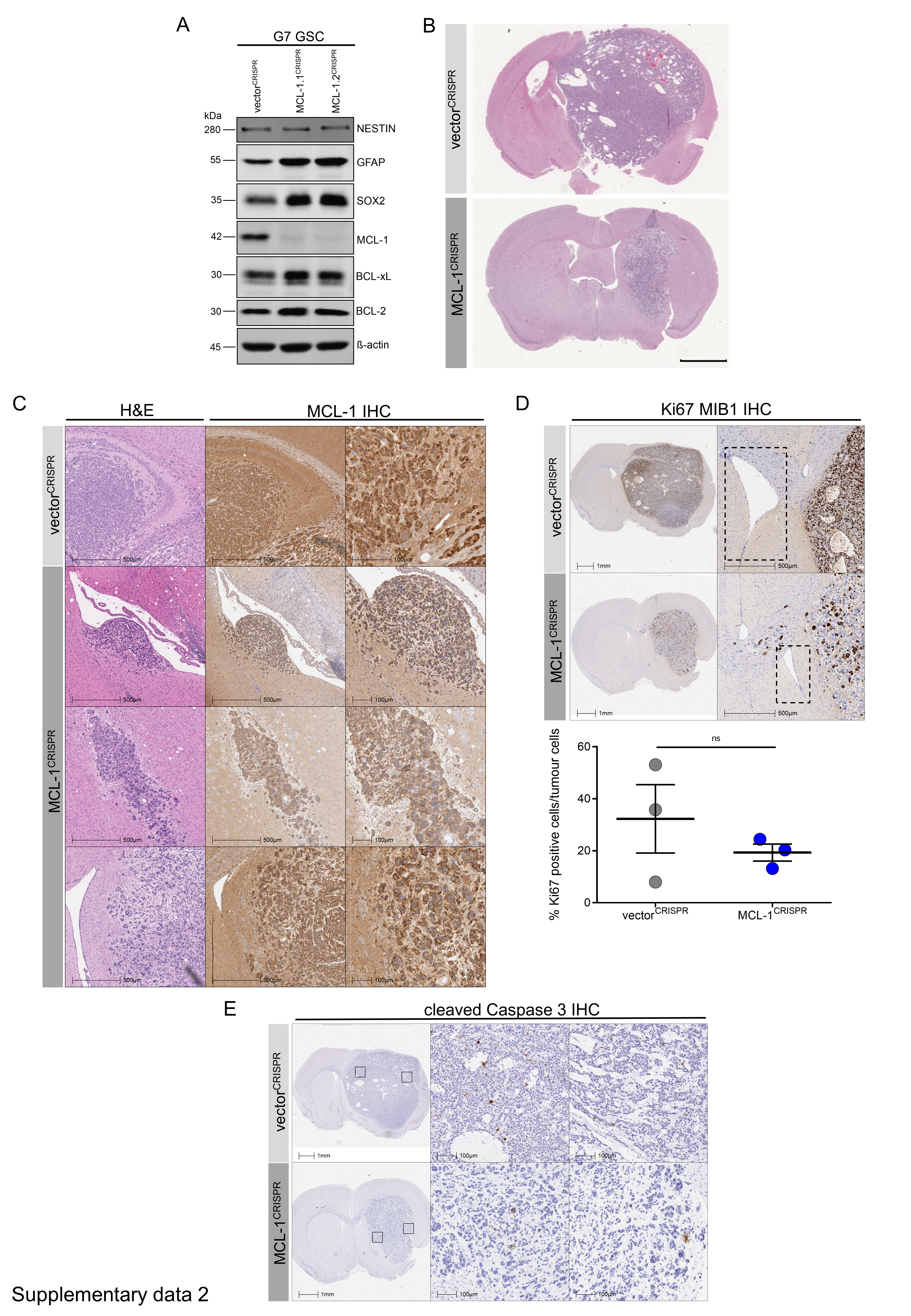

Supplement: Supplementary file 2 — Suppemental Figure 2 [file 41418_2022_1001_MOESM2_ESM.tif]

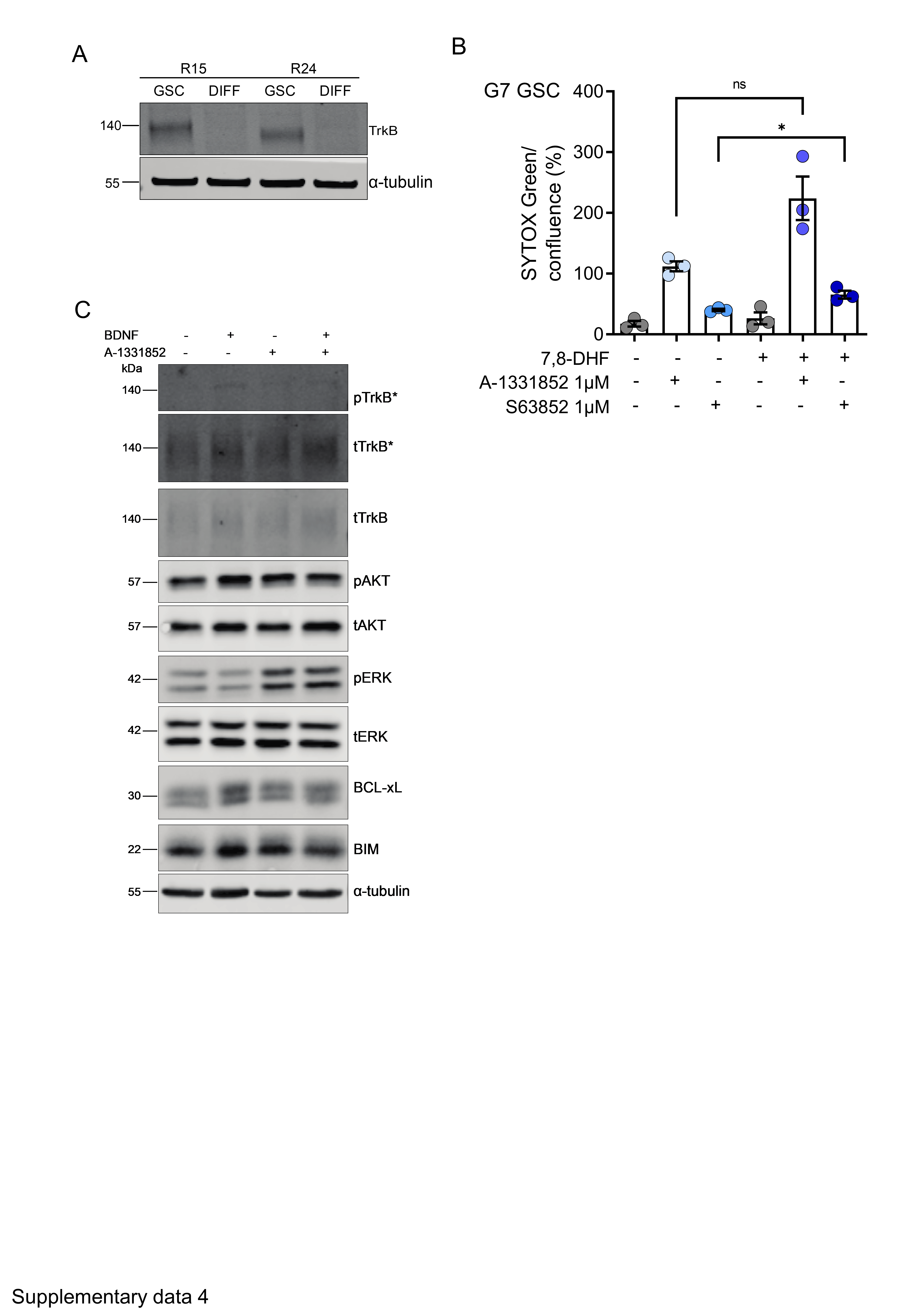

Supplement: Supplementary file 3 — Suppemental Figure 4 [file 41418_2022_1001_MOESM3_ESM.tif]

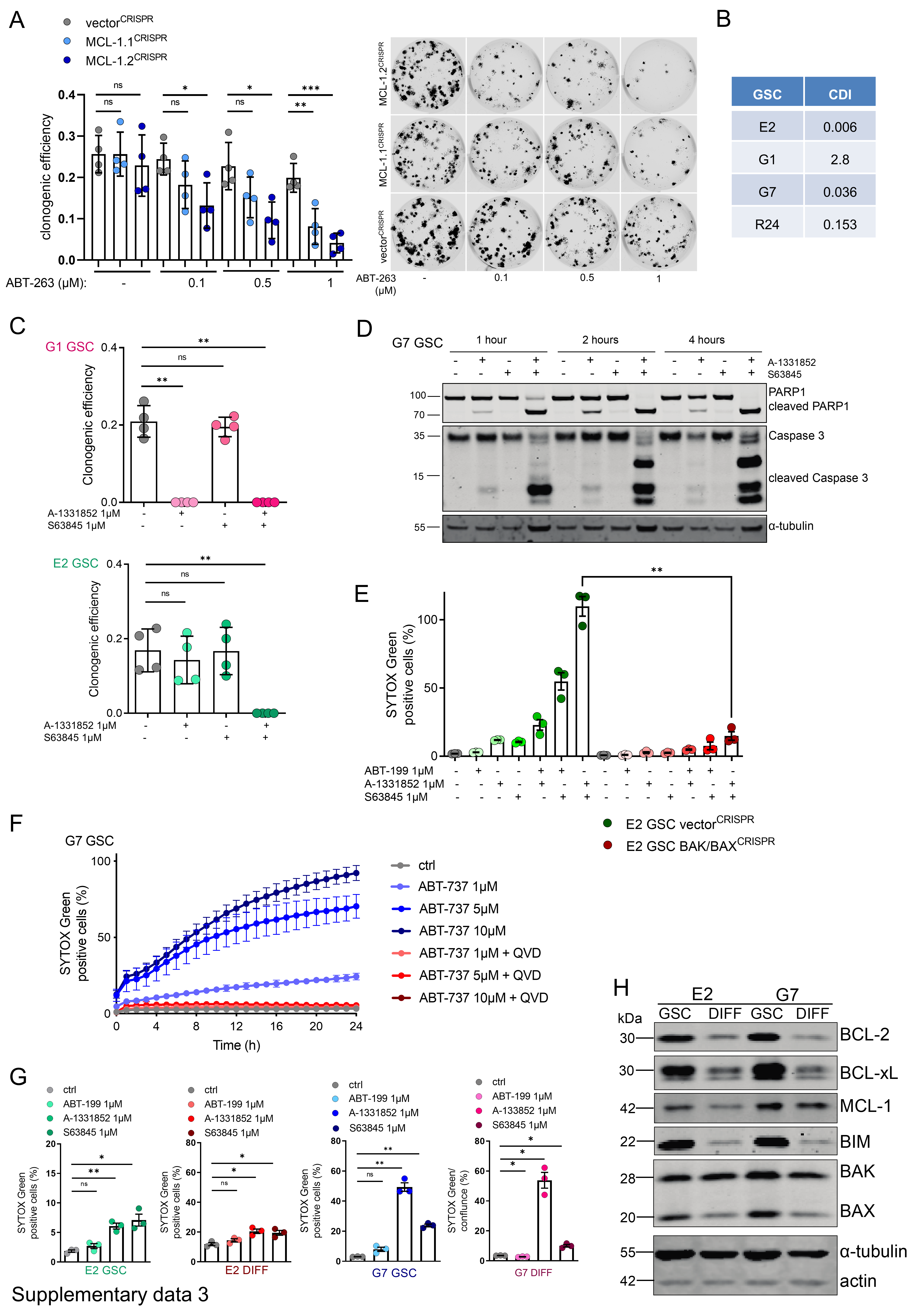

Supplement: Supplementary file 4 — Suppemental Figure 3 [file 41418_2022_1001_MOESM4_ESM.tif]

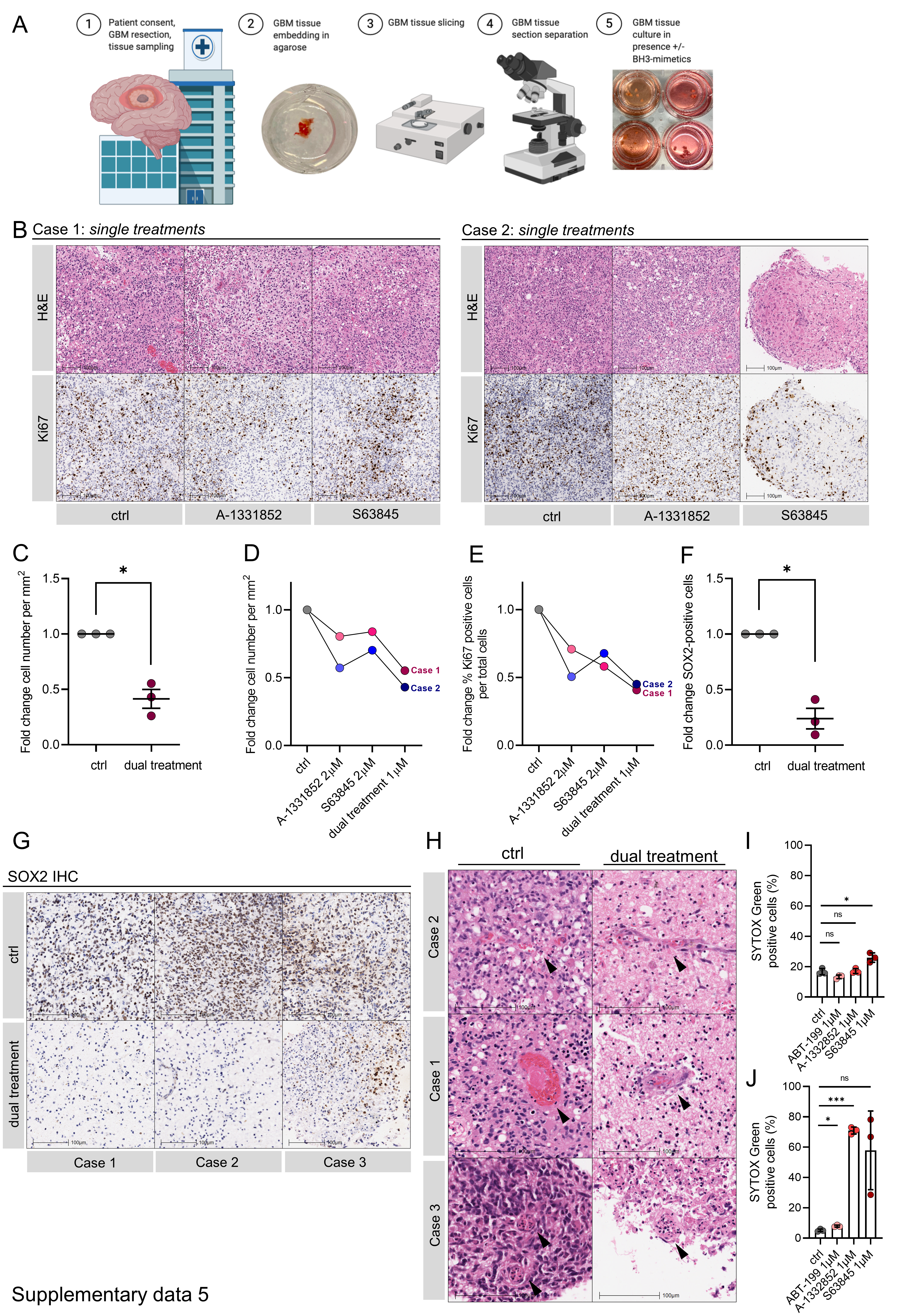

Supplement: Supplementary file 5 — Suppemental Figure 5 [file 41418_2022_1001_MOESM5_ESM.tif]

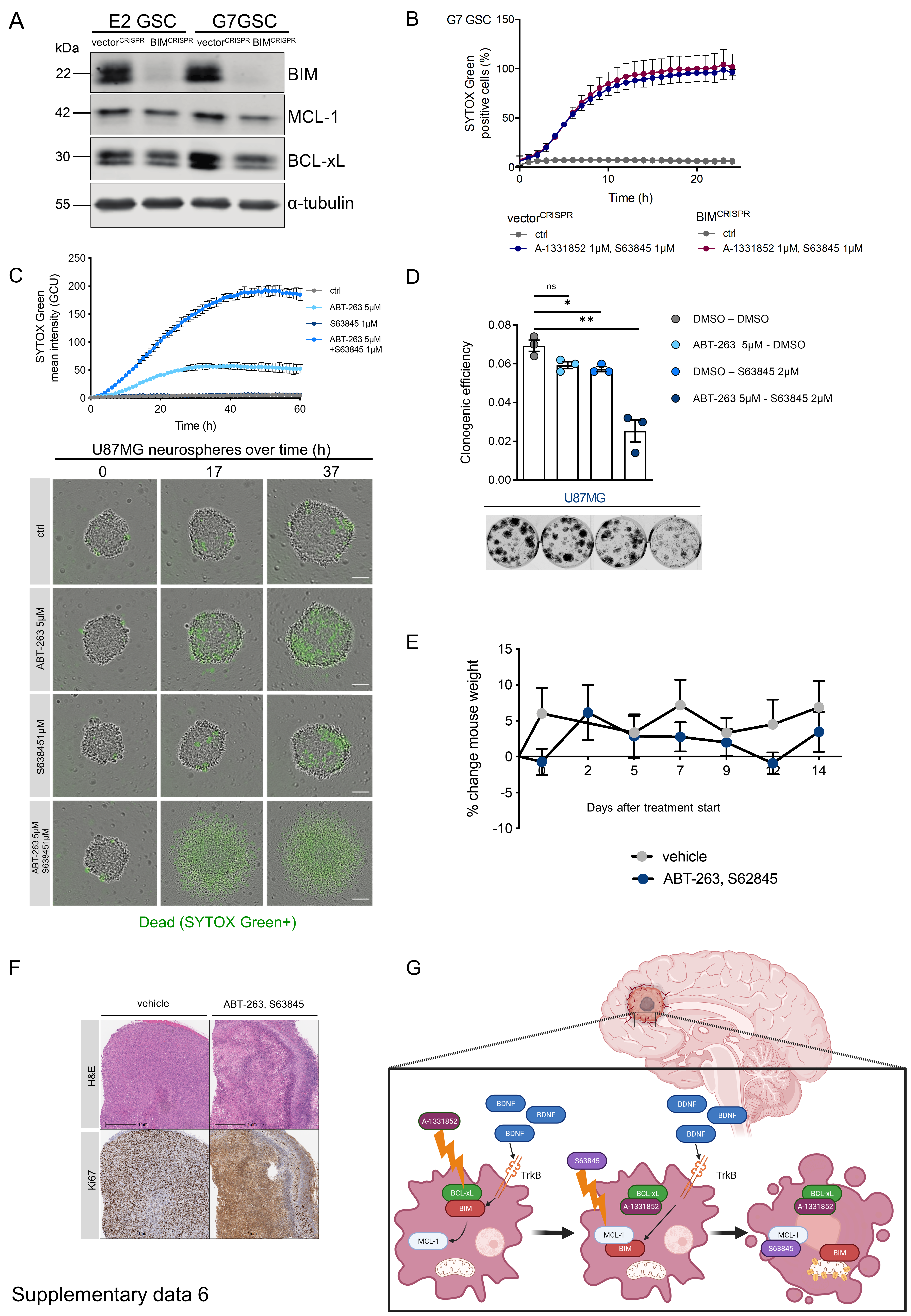

Supplement: Supplementary file 6 — Suppemental Figure 6 [file 41418_2022_1001_MOESM6_ESM.tif]
