# Supplementary material for: Increased apoptotic sensitivity of glioblastoma enables therapeutic targeting by BH3-mimetics
Source: Cell Death Differ. 2022 Apr 26;29(10):2089–104. doi: 10.1038/s41418-022-01001-3 (PMC9525582; doi:10.1038/s41418-022-01001-3)

Source data

Figure 1C

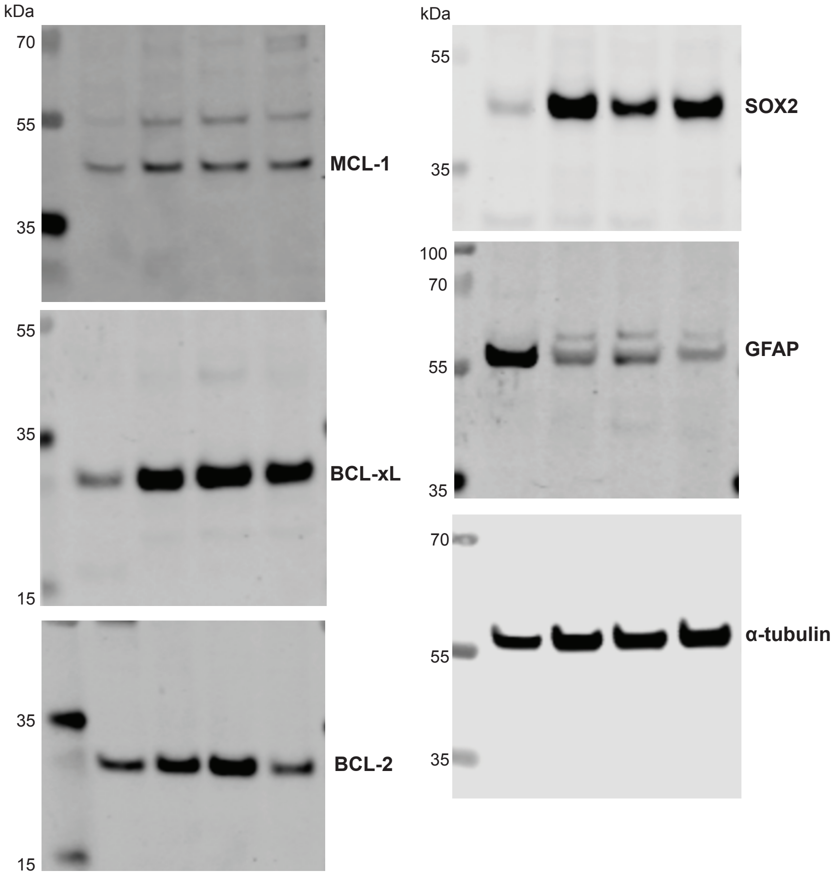

Figure 3A

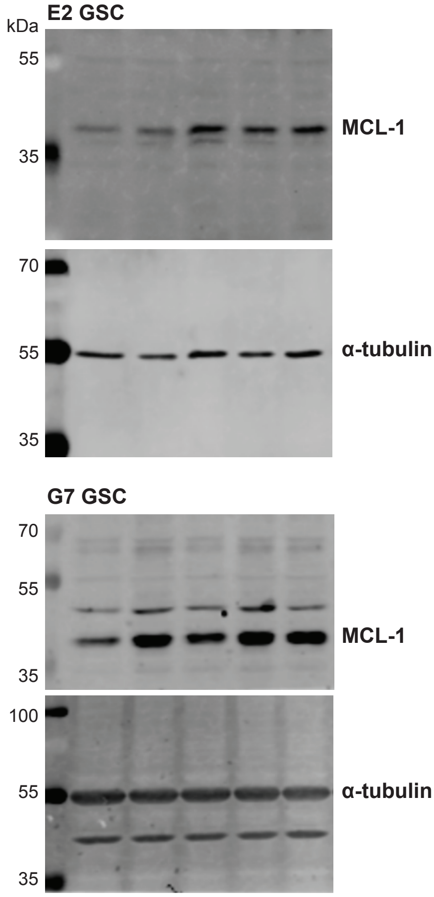

Figure 3E

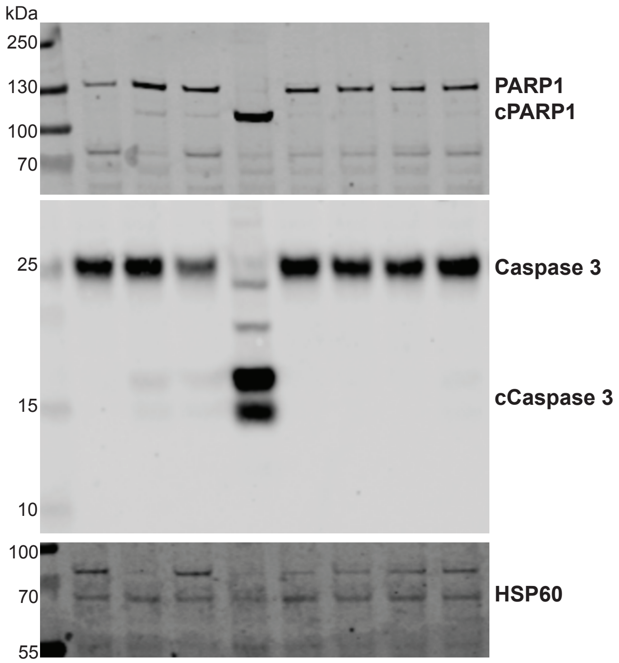

Koessinger et al., Increased apoptotic priming of glioblastoma enables therapeutic targeting by BH3-mimetics.

Source data

Figure 4B

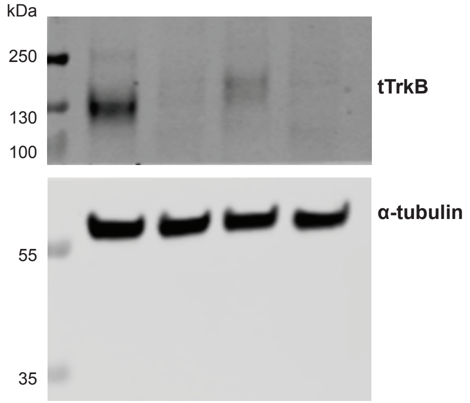

Figure 4E

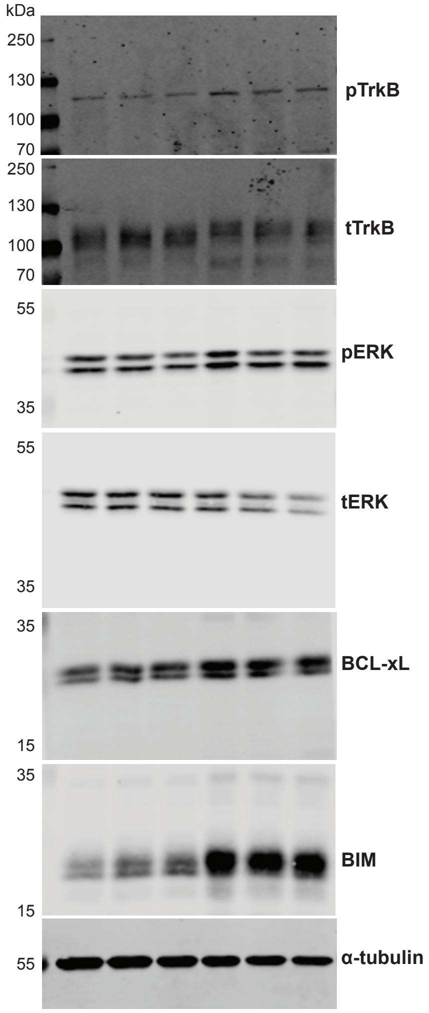

Figure 6A

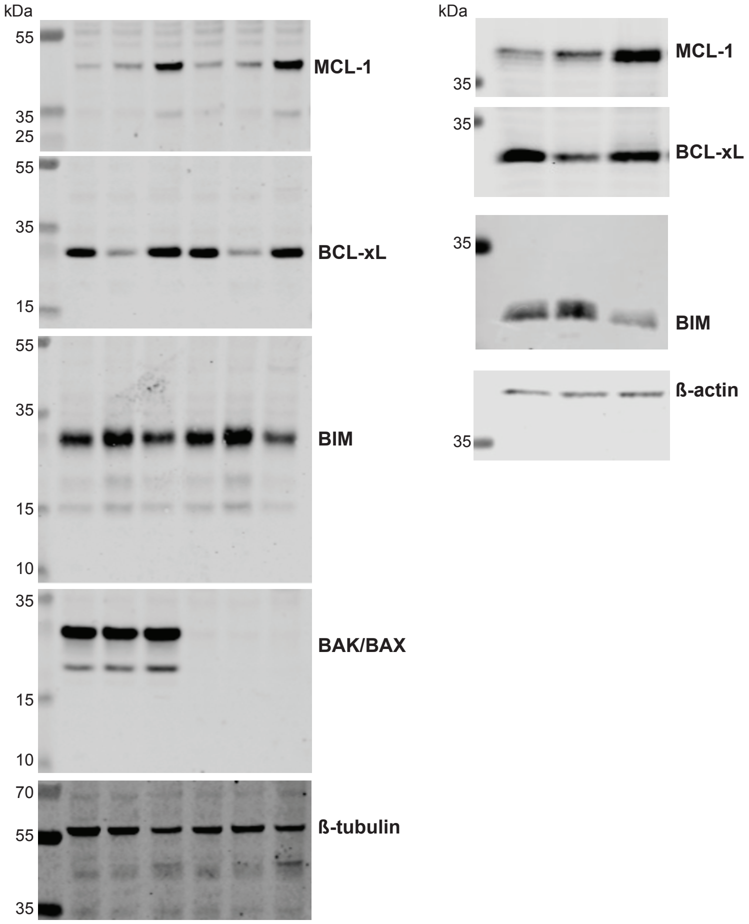

Figure 6B

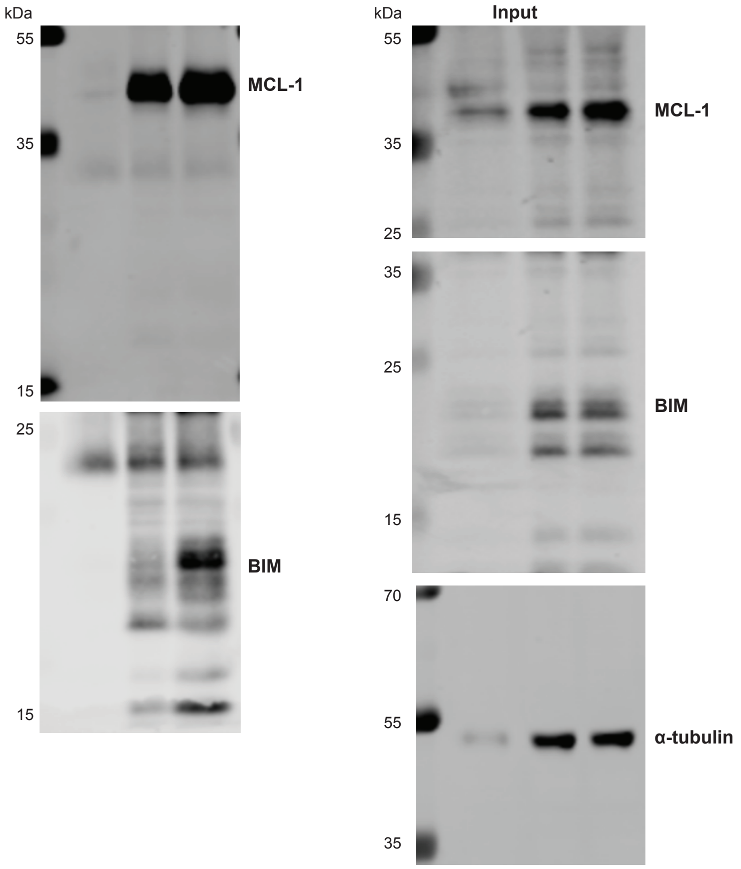

Koessinger et al., Increased apoptotic priming of glioblastoma enables therapeutic targeting by BH3-mimetics.

Source data

Supplementary Figure 2A

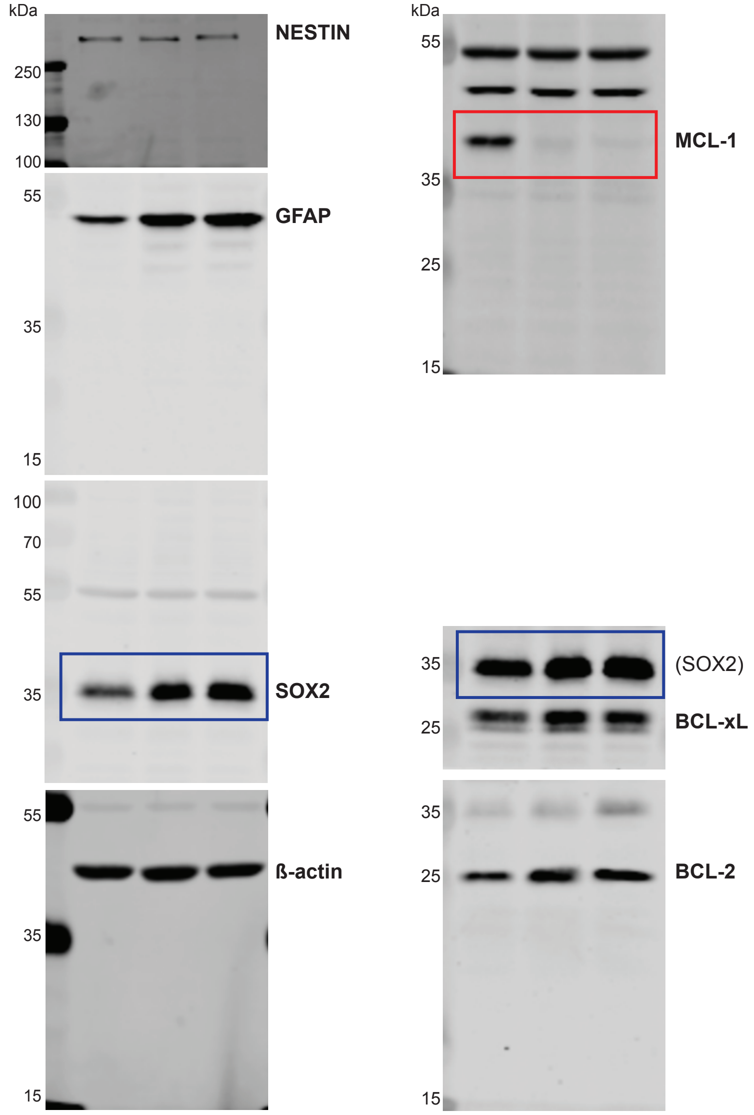

Supplementary Figure 3H

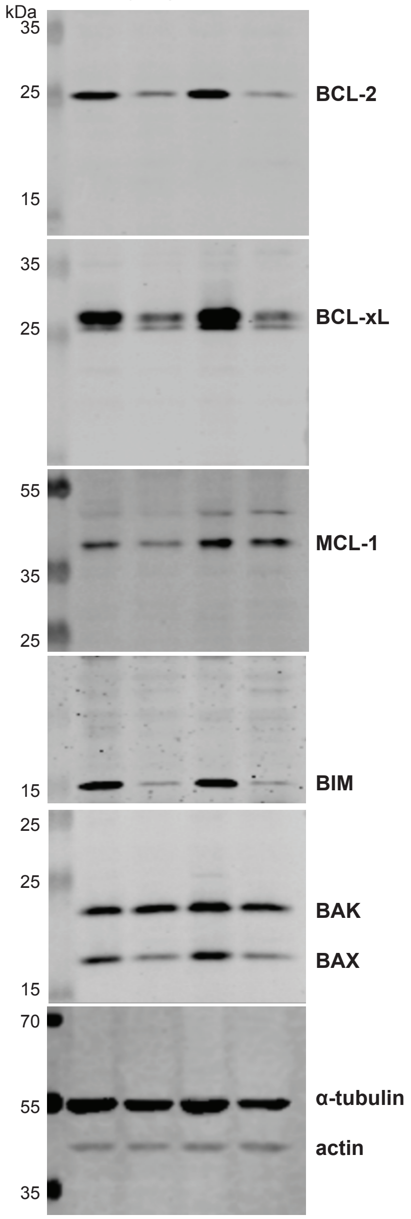

Supplementary Figure 3D

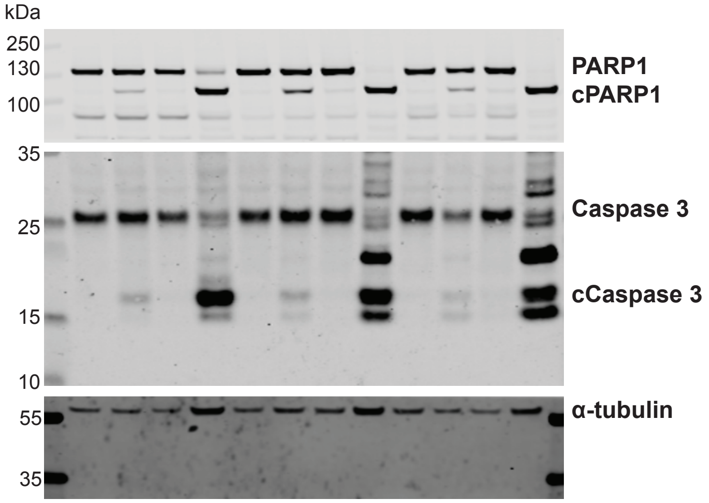

Koessinger et al., Increased apoptotic priming of glioblastoma enables therapeutic targeting by BH3-mimetics.

Source data

Supplementary Figure 4A

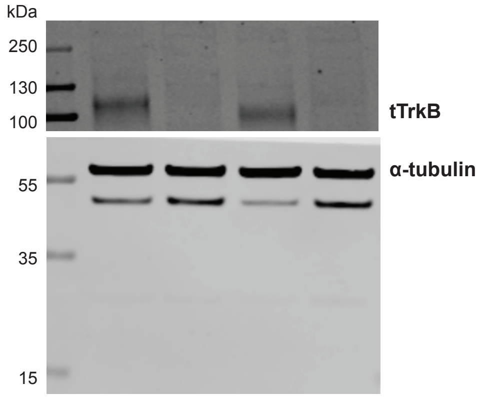

Supplementary Figure 4C

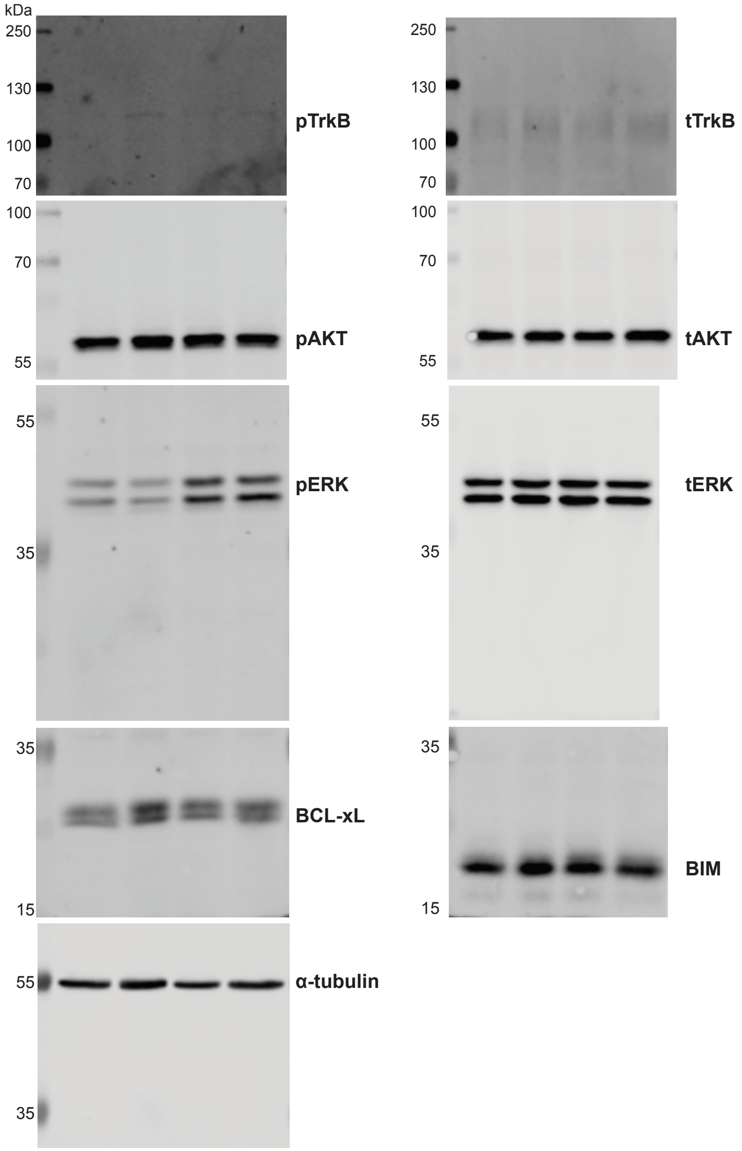

Supplementary Figure 6A

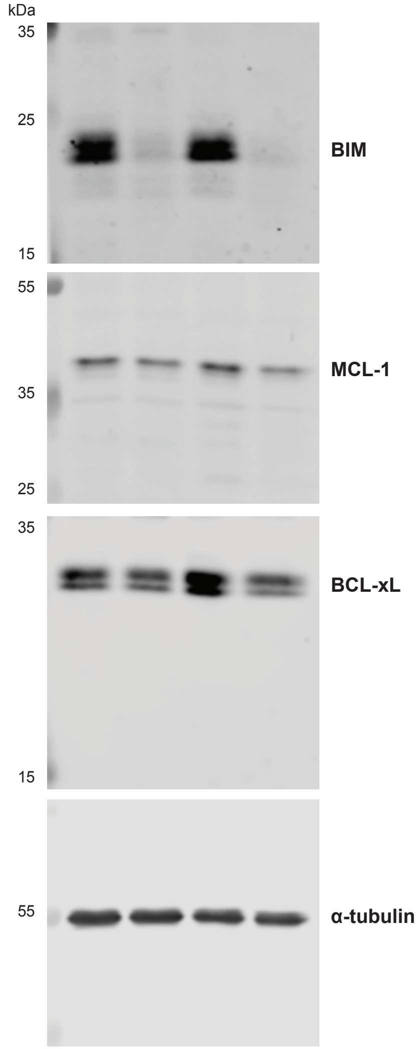

Supplement: Supplementary file 7 — Source data - uncropped blots [file 41418_2022_1001_MOESM7_ESM.pdf]
