# Supplementary material for: Increased apoptotic sensitivity of glioblastoma enables therapeutic targeting by BH3-mimetics
Source: Cell Death Differ. 2022 Apr 26;29(10):2089–104. doi: 10.1038/s41418-022-01001-3 (PMC9525582; doi:10.1038/s41418-022-01001-3)
Supplement: Supplementary file 8 — Supplemental Figure Legends [file 41418_2022_1001_MOESM8_ESM.docx]

**Supplementary Figure 1. Relevant to figure 1.**

Single RNA-sequencing of 28 freshly resected GBM specimens with quantification of BCL-2 family genes in correlation with cell cycle distribution in G2M and G1S phase (**A**) and comparison of recognised pattern with the Neftel gene expression classification (**B**). (**C**) Corresponding representative H&E images of GBM tumour and margin samples shown in lower magnification than in Figure 1D. (**D**) *BCL-xL* and *MCL-1* mRNA expression from the publicly available REMBRANDT GBM microarray dataset. Data plotted for different glioma subtypes and normal brain tissue. Error bars represent mean +/-SEM (**p*=0.0118, *****p*<0.0001) Welch’s test. (**E**) Clonogenic survival of G7 GSC treated with radiation alone (red line) and following incubation of ABT-737 with indicated doses 24 hours prior to radiation; SER (sensitiser enhancement ratio) and p-values given in adjacent table. (**F**) E2 and G7 GSC were treated with the indicated drugs for 48 hours and analysed for cell viability using an IncuCyte imager and SYTOX Green exclusion. Percentage cell death was calculated by normalising against maximal cell death as described before. Error bars represent mean +/-SEM from *n*=3 independent experiments (E2 ns *p*≥0.6601, G7 ns *p*≥0.1585) Welch’s test.

**Supplementary Figure 2. Relevant to figure 2.**

(**A**) Immunoblotting of G7 GSC vector^CRISPR^, MCL-1.1^CRISPR^ and MCL-1.2^CRISPR^ for BCL-2 family proteins, cell-line specific neural stem cell marker (SOX2, NESTIN) and astrocyte lineage differentiation marker GFAP. β-actin served as loading control. (**B**) Representative H&E images of orthotopic G7 GSC iRFP vector^CRISPR^ and MCL-1^CRISPR^ xenografts at end point (corresponding to Figure 2B). Scale bar = 100µm. (**C**) Representative images of H&E and MCL-1 IHC of G7 GSC iRFP vector^CRISPR^ and three MCL-1^CRISPR^ xenografts at end point. (**D**) Representative images of human specific Ki67 Mib1 IHC in G7 GSC iRFP vector^CRISPR^ and MCL-1^CRISPR^ xenografts at end point with quantification of percentage Ki67 positive cells within the tumours. Error bars represent mean +/-SEM (ns *p*=0.4292). Box with dashed line indicates lateral ventrical with subventricular zone including the murine neurogenic niche as internal negative control. (**E**) Representative images of cleaved Caspase 3 IHC in G7 GSC iRFP vector^CRISPR^ and MCL-1^CRISPR^ xenografts at end point.

**Supplementary Figure 3. Relevant to figure 3.**

(**A**) Clonogenic survival assay of G7 GSC iRFP vector^CRISPR^ vs. MCL-1.1^CRISPR^ and MCL-1.2^CRISPR^ treated with indicated drugs 16 hours after plating 250 cells per well. Colonies counted manually after 14 days. Error bars represent mean +/-SD from *n*=4 independent experiments (ns *p*≥0.0922, **p*≤0.018, ***p*≤0.0059, ****p*=0.0005). Representative images scanned on LICOR imager. (**B**) Table of GSC and their coefficient of drug interaction (CDI) for dual A-1331852 and S63845 inhibition (1µM each) compared to single drug treatment; CDI <0.7 indicates a significantly synergistic effect (71). (**C**) Clonogenic survival assay of E2 and G1 GSC iRFP treated with indicated drugs 16 hours after plating 250 cells per well. Colonies counted manually after 14 days. Error bars represent mean +/-SEM from *n*=4 independent experiments (E2 ns p=0.573, ***p*=0.0099, G1 ns *p*=0.5774, ***p*=0.002) Welch’s test. (**D**) G7 GSC were treated with DMSO (-), A-1331852 and S63845 for indicated times, harvested and protein expression was analysed by immunoblot. α-tubulin served as loading control. Representative image from three independent experiments. (**E**) E2 GSC vector^CRISPR^ and BAK/BAX^CRISPR^ treated with indicated drugs for 48 hours and analysed for cell viability using an IncuCyte imager and SYTOX Green exclusion. Percentage cell death was calculated by normalising against maximal cell death as described in Figure 1B. Error bars represent mean +/-SEM from *n*=3 independent experiments (***p*=0.0019) (**F**) G7 GSC treated with indicated drugs (+/-QVD 10µM) for 24 hours and analysed for cell viability using an IncuCyte imager and SYTOX Green exclusion. Percentage cell death was calculated by normalising against maximal cell death as described in Figure 1B. Error bars represent mean +/-SEM from *n*=3 independent experiments. (**G**) E2 and G7 GSC with paired DIFF cells treated with indicated drugs for 24 hours and analysed for cell viability using an IncuCyte imager and SYTOX Green exclusion. Percentage cell death was calculated by normalising against maximal cell death as described in Figure 1B. Error bars represent mean +/-SEM from *n*=3 independent experiments (E2 GSC ns *p*=0.1765, **p*=0.0307, ***p*=0.0066, E2 DIFF ns *p*=0.167, **p*≤0.0223, G7 GSC ns *p*=0.0708, **p*≤0.0037, G7 DIFF **p*≤0.0167). (**H**) Immunoblot of E2 and G7 GSC with paired DIFF cells for BCL-2 family proteins. α-tubulin served as loading control. Representative image from *n*=2 independent experiments.

**Supplementary Figure 4. Relevant to figure 4.**

(**A**) Immunoblot of TrkB in R15 and R24 GSC compared with paired DIFF cells. α-tubulin served as loading control. Representative image from *n*=2 independent experiments. (**B**) G7 GSC treated with indicated drugs for 24h hours in full stem-cell medium (including EGF and FGF) and analysed for cell viability using an IncuCyte imager and SYTOX Green exclusion. Error bars represent mean +/-SEM from *n*=3 independent experiments (ns *p*=0.0817, **p*=0.05) Welch’s test. (**C**) G7 DIFF were treated with A-1331852 2μM +/- BDNF (100ng/mL) for 1 hour, harvested and protein expression was analysed by immunoblot (*indicates high exposure). α-tubulin served as loading control. Representative image from *n*=3 independent experiments.

**Supplementary Figure 5. Relevant to figure 5.**

(**A**) Schematic model of fresh GBM patient tissue collection and processing. (**B**) Representative H&E and Ki67 IHC images of GBM case 1 and 2 treated with single inhibitors A-1331852 2μM or S63845 2μM for 72 hours. In both cases H&E control images were used from the same area as shown in Figure 5A,B in different magnification. (**C**) Quantification of cell reduction in GBM case 1-3 normalised to DMSO-treated control. Error bars represent mean +/-SEM (**p*=0.0204) Welch’s test. (**D,E**) Change of cellularity and percentage Ki67 positive cells/tumour cell count in GBM case 1 and 2 treated with the indicated drugs for 72 hours. (**F**) Quantification of SOX2 positive cell reduction in GBM case 1-3 normalised to DMSO-treated control. Error bars represent mean +/-SEM (**p*=0.0145) Welch’s test. (**G**) Representative images of SOX2 IHC in GBM case 1-3. (**H**) Representative H&E images of GBM case 1-3, arrows indicate intratumoural vessels. (**I,J**) hAS and hNS were treated with indicated drugs for 24 hours and analysed for cell viability using an IncuCyte imager and SYTOX Green exclusion. Percentage cell death was calculated by normalising against maximal cell death as described in Figure 1B. (hAS: error bars represent mean +/-SEM from *n*=3 independent experiments, ns, *p*=0.072, **p*=0.0104, ****p*=0.0001; hNS: error bars represent mean +/-SEM from three replicates in *n*=1, ns, *p*≥0.0813, **p*=0.0178) Welch’s test.

**Supplementary Figure 6. Relevant to figure 6.**

(**A**) Immunoblot of E2 and G7 GSC vector^CRISPR^ and BIM^CRISPR^ for BCL-xL, MCL-1 and BIM. Representative images from *n*=2 independent experiments. α-tubulin served as loading control. (**B**) G7 GSC vector^CRISPR^ and BIM^CRISPR^ treated with DMSO or indicated combination for 24 hours and analysed for cell viability using an IncuCyte imager and SYTOX Green exclusion. Error bars represent mean +/-SD from *n*=3 independent experiments. (**C**) Representative images of U87MG neurospheres in stem-cell medium under treatment with indicated drugs over a total of 60 hours. Quantification of neurosphere treatment for cell viability using an IncuCyte imager and SYTOX Green exclusion given as mean signal intensity (GCU). One representative of *n*=3 independent experiments shown. Error bars represent mean +/-SD. (**D**) Clonogenic survival assay of U87MG. Treatment commenced 16 hours after plating 250 cells/well with either DMSO or ABT-263 5μM for 24 hours followed by drug washout and treatment pause for 24 hours and S63845 2μM for 24 hours. Alternating treatment was continued over the experimental period of 14 days. Colonies were counted manually. Quantification of one representative of *n*=2 independent experiments shown, error bars represent mean +/-SEM (ns, *p*=0.0538, **p*=0.037, ***p*=0.0065). Representative images of a replicate in one independent repeat scanned on LICOR imager. (**E**) Percent weight change of mice (vehicle *n*=6, ABT-236, S63845 *n*=7) during the drug treatment period of the experiment in Figure 1G,H. (**F**) Representative H&E and Ki67 IHC images of U87MG xenografts treated with vehicle or alternating ABT-263 and S63845 therapy at end point. (**G**) Summary schematic.
