# Supplementary material for: Increased apoptotic sensitivity of glioblastoma enables therapeutic targeting by BH3-mimetics
Source: Cell Death Differ. 2022 Apr 26;29(10):2089–104. doi: 10.1038/s41418-022-01001-3 (PMC9525582; doi:10.1038/s41418-022-01001-3)
Supplement: Supplementary file 9 — Change of authorship form [file 41418_2022_1001_MOESM9_ESM.pdf]

SPRINGER NATURE

Change of authorship request form - Journals (pre-acceptance)

Important information. Please read.

- This form should be used by authors to request any change in authorship (adding/deleting authors) including changes in corresponding authors. This form should not be used for name changes. Please fully complete all sections. Use black ink and block capitals and provide each author's full name with the given name first followed by the family name.

- By signing this declaration, all authors guarantee that the order of the authors are in accordance with their scientific contribution, if applicable as different conventions apply per discipline, and that only authors have been added who made a meaningful contribution to the work.

- Please note, in author collaborations where there is formal agreement for representing the collaboration, it is sufficient for the representative or legal guarantor (usually the corresponding author) to complete and sign the Authorship Change Form on behalf of all authors, **next to the added/removed author(s)**. **(Complete Section 3, followed by Section 6.)**
  In author collaborations where there is no formal agreement for representing the collaboration and **there are more than 10 authors**, one may sign for all, provided the signer appends correspondence that attests that each of the authors have agreed to the change **and the added/removed authors sign the form**. **(Complete Section 3, followed by Section 6.)**

- Please note, we cannot investigate or mediate any authorship disputes. If you are unable to obtain agreement from all authors (including those who you wish to be removed) you must refer the matter to your institution(s) for investigation. Please inform us if you need to do this.

- If you are not able to return a fully completed form within **30 days** of the date that it was sent to the author requesting the change, we may have to withdraw your manuscript. We cannot publish manuscripts where authorship has not been agreed by all authors (including those who have been removed).

- Incomplete forms will be rejected.

- Please return/upload this form, fully completed, to the Journals Editorial Office. The Journal and/or Publisher will consider the information you have provided to decide whether to approve the proposed change in authorship. We may decide to contact your institution for more information or undertake a further investigation, if appropriate, before making a final decision.

SPRINGER NATURE Springer Nature is one of the world's leading global research, educational and professional publishers, created in May 2015 through the combination of Nature Publishing Group, Palgrave Macmillan, Macmillan Education and Springer Science+Business Media.

SPRINGER NATURE

Change of authorship request form - Journals (pre-acceptance)

Section 1: Please provide the current title of manuscript

Manuscript ID no.: CDD 21 0793RR

Title: INCREASED APOPTOTIC SENSITIVITY OF GLIOBLASTOMA ENABLES THERAPEUTIC TARGETING BY BH3-MIMETICS

Section 2: Please provide the previous authorship, in the order shown on the manuscript before the changes were introduced. Please indicate the corresponding author by adding (CA) behind the name.

	First name(s)	Family name	ORCID or SCOPUS id, if available
1st author	ANNA	KOESSINGER	
2nd author	DOMINIK	KOESSINGER	
3rd author	KEVIN	KINCH	
4th author	LAURA	MARTINEZ ESCARDO	
5th author	NIKKI	PAUUL	
6th author	YASSMIN	ELMASRY	
7th author	GAURAV	MALVIYA	
8th author	CATHERINE	CLOIX	
9th author	KIRSTEEN	CAMPBELL	
10th author	FLORIAN	BOCK	

Please use an additional sheet if there are more than 10 authors.

SPRINGER NATURE Springer Nature is one of the world's leading global research, educational and professional publishers, created in May 2015 through the combination of Nature Publishing Group, Palgrave Macmillan, Macmillan Education and Springer Science+Business Media.

SPRINGER NATURE

Change of authorship request form - Journals (pre-acceptance)

Section 1: Please provide the current title of manuscript

Manuscript ID no.: CDD 2[illegible] 0793 RR

Title: INCREASED APOPTOTIC SENSITIVITY OF GLIOBLASTOMA ENABLES THERAPEUTIC TARGETING BY BH3-MIMETICS

Section 2: Please provide the previous authorship, in the order shown on the manuscript before the changes were introduced. Please indicate the corresponding author by adding (CA) behind the name.

	First name(s)	Family name	ORCID or SCOPUS id, if available
1st author	JIM [illegible]	OPREY	
2nd author	KATRINA	STEVENSON	
3rd author	COLIN	NIXON	
4th author	MARK	JACKSON	
5th author	GABRIEL	ICHIM	
6th author	WILLIAM	STEWART	
7th author	KAREN	BLYTH	
8th author	KEVIN	RYAN	
9th author	ANT	CHALMERS	
10th author	JIM	NORMAN	

Please use an additional sheet if there are more than 10 authors.

SPRINGER NATURE Springer Nature is one of the world's leading global research, educational and professional publishers, created in May 2015 through the combination of Nature Publishing Group, Palgrave Macmillan, Macmillan Education and Springer Science+Business Media.

SPRINGER NATURE

Change of authorship request form - Journals (pre-acceptance)

Section 1: Please provide the current title of manuscript

Manuscript ID no.: CDD210793RR

Title: INCREASED APOPTOTIC SENSITIVITY OF GLIOBLASTOMA ENABLES THERAPEUTIC TARGETING BY BH3-MIMETICS

Section 2: Please provide the previous authorship, in the order shown on the manuscript before the changes were introduced. Please indicate the corresponding author by adding (CA) behind the name.

	First name(s)	Family name	ORCID or SCOPUS id, if available
2\ ~~1st~~ author	STEPHEN	TAIT (CA)	
2nd author			
3rd author			
4th author			
5th author			
6th author			
7th author			
8th author			
9th author			
10th author			

Please use an additional sheet if there are more than 10 authors.

SPRINGER NATURE Springer Nature is one of the world's leading global research, educational and professional publishers, created in May 2015 through the combination of Nature Publishing Group, Palgrave Macmillan, Macmillan Education and Springer Science+Business Media.

SPRINGER NATURE

Change of authorship request form - Journals (pre-acceptance)

Section 5: Author contribution, Acknowledgement and Disclosures. Please use this section to provide a new disclosure statement and, if appropriate, acknowledge any contributors who have been removed as authors and ensure you state what contribution any new authors made (if applicable per the journal or book (series) policy). Please ensure these are updated in your manuscript - after approval of the change(s) - as our production department will not transfer the information in this form to your manuscript.

New acknowledgements:

First and foremost, our special gratitude goes to the GBM patients who agreed to make their tumour specimens available for research. Special thanks to Mary Fraser, Dr. Alexandru Stan, Dr. Zoltan Hanzely and the Neuropathology Department of the QUEH in Glasgow, who obtained patient consent and provided support with GBM tissue sampling. Further, we would like to thank the Core Services at the Cancer Research UK Beatson Institute with particular thanks to Gemma Thomson, David Strachan, the Biological Services Unit and Histology. We thank Prof. Colin Watts for generous sharing of the patient-derived GBM cell lines and Prof. Steven Pollard for providing the human neural stem cells and astrocytes, which were made available through the Glioma Cellular Genetics Resource funded by Cancer Research UK. Many thanks to Dr. Jayanthi Anand and Dr. Dimitris Athineos for their invaluable support with the animal work. Many thanks to members of the Tait laboratory for discussion and input, and Catherine Winchester for critical reading and assistance in the preparation of this manuscript. Figures were created with BioRender.com. Funding:

This work was supported by CRUK core funding to the Beatson Institute (A17196) to C.N., K.B. (A29799) to K.B. K.C., (A17196/A31287) to N.R.P., (A22903) to K.M.R. and J.O'P., and (A18277) to J.C.N., a CRUK Programme Foundation Award (C40872/A20145) to S.W.T., CRUK Clinical Research Fellowship (A23220) to A.L.K., funding by the University of Glasgow to D.K. and A.J.C. and funding by the Beatson Cancer Charity and Cancer Research UK RadNet Centre Glasgow (A28803) to K.S., S.D. and K.S.. L.M.-E. was funded by the by Erasmus+ Program and Short Research stay fellowship for trainees by Universitat Autònoma de Barcelona and A.A. by Prostate Cancer UK RIA17-ST2-002. DHH is funded by the Else Kröner-Fresenius Foundation. The work is part of the MEPHISTO project, funded by BMBF (iGerman Ministry of Education and Research, project number: 031L0260B).

New Disclosures (financial and non-financial interests, funding):

None

New Author Contributions statement (if applicable per the journal policy):

A.L.K. and S.W.G.T. conceived the study, designed the work plan and wrote the manuscript. A.L.K. performed the majority of the experiments. C.C., D.K., L.M.-E., J.O'P. and F.J.B. performed particular experiments or acquired data and provided technical support. D.H.H. conducted and provided the spatial transcriptomics analysis. N.R.P. performed IF imaging and analysis. D.K., K.S., C.C., A.A., C.N. and G.M. provided assistance with the in vivo models and related imaging. K.K. and W.S. supervised and supported patient specimen collection. A.J.C. provided the RNAseq dataset. Y.E., D.K. and M.R.J. performed and supported data analysis. Expertise and critical input as well as review of the manuscript was given by K.J.C., O.S., G.I., K.B., A.J.C., K.M.R. and J.C.N.

State 'Not applicable' if there are no new authors.

SPRINGER NATURE Springer Nature is one of the world's leading global research, educational and professional publishers, created in May 2015 through the combination of Nature Publishing Group, Palgrave Macmillan, Macmillan Education and Springer Science+Business Media.

SPRINGER NATURE — Change of authorship request form - Journals (pre-acceptance)

Section 3: Please provide a justification for change. Please use this section to explain your reasons for changing the authorship of your manuscript, e.g. what necessitated the change in authorship? Please refer to the (journal) policy pages for more information about authorship. Please explain why omitted authors were not originally included and/or why authors were removed on the submitted manuscript.

NEW AUTORS ADDED BECAUSE THEY DID EXPTS. FOR REVISED VERSION

Section 4: Proposed new authorship. Please provide your new authorship list in the order you would like it to appear on the manuscript. Please indicate the corresponding author by adding (CA) behind the name. If the Corresponding Author has changed, please indicate the reason under section 3.

	First name(s)	Family name (this name will appear in full on the final publication and will be searchable in various abstract and indexing databases)	Affiliated institute	E-mail address
1st author	ANNA	KOESSINGER	UNI GLASGOW	anna.koessinger@glasgow.ac.uk
2nd author	CAT	CLOIX	"	cat.cloix@ "
3rd author	DOMINIK	KOESSINGER	"	dominik.koessinger@ "
4th author	DIETER	HEILAN	UNI FREIBURG	dieter.heiland@uni-freiburg.de
5th author	FLO	BOCK	UNI GLASGOW	florian.bock@glasgow.ac.uk
6th author	KAREN	STRATHDEE	"	karen.strathdee@ "
7th author	KEV	KINCH	"	kevin.kinch@ "
8th author	LAURA	ESCARD	"	laura.escardo@ "
9th author	NIKKI, ~~[illegible]~~	PAUL	"	nikki.paul@ "
10th author	COLIN	NIXON	"	colin.nixon@ "

Please use an additional sheet if there are more than 10 authors.

SPRINGER NATURE Springer Nature is one of the world's leading global research, educational and professional publishers, created in May 2015 through the combination of Nature Publishing Group, Palgrave Macmillan, Macmillan Education and Springer Science+Business Media.

SPRINGER NATURE Change of authorship request form - Journals (pre-acceptance)

Section 3: Please provide a justification for change. Please use this section to explain your reasons for changing the authorship of your manuscript, e.g. what necessitated the change in authorship? Please refer to the (journal) policy pages for more information about authorship. Please explain why omitted authors were not originally included and/or why authors were removed on the submitted manuscript.

SEE FIRST SHEET.

Section 4: Proposed new authorship. Please provide your new authorship list in the order you would like it to appear on the manuscript. Please indicate the corresponding author by adding (CA) behind the name. <u>If the Corresponding Author has changed, please indicate the reason under section 3</u>.

	First name(s)	Family name (this name will appear in full on the final publication and will be searchable in various abstract and indexing databases)	Affiliated institute	E-mail address
1st author	GAURAV	MALVIYA	UNI GLASGOW	gaurav.malviya@glasgow.ac.uk
2nd author	MARK	JACKSON	"	mark.jackson@ "
3rd author	KIRSTEEN	CAMPBELL	"	kirsteen.campbell@ "
4th author	KATRINA	STEVENSON	"	katrina.stevenson@ "
5th author	SANDEEP	DAVIS	"	sandeep.davis@ "
6th author	YASSMIN	ELMASRY	"	yassmin.elmasry@ "
7th author	ASMA	AHMED	"	asma.ahmed@glasgow.ac.uk
8th author	JIM	OPREY	"	jim.oprey@glasgow.ac.uk
9th author	GABRIEL	ICHIM	CRCL LYON	gabriel.ichim@crcl.fr
10th author	OLIVER	SCHNELL	UNI FREIBURG	oliver.schnell@uni-freiburg.de

Please use an additional sheet if there are more than 10 authors.

SPRINGER NATURE Springer Nature is one of the world's leading global research, educational and professional publishers, created in May 2015 through the combination of Nature Publishing Group, Palgrave Macmillan, Macmillan Education and Springer Science+Business Media.

SPRINGER NATURE # Change of authorship request form - Journals (pre-acceptance)

Section 3: Please provide a justification for change. Please use this section to explain your reasons for changing the authorship of your manuscript, e.g. what necessitated the change in authorship? Please refer to the (journal) policy pages for more information about authorship. Please explain why omitted authors were not originally included and/or why authors were removed on the submitted manuscript.

SEE FIRST SHEET

Section 4: Proposed new authorship. Please provide your new authorship list in the order you would like it to appear on the manuscript. Please indicate the corresponding author by adding (CA) behind the name. <u>If the Corresponding Author has changed, please indicate the reason under section 3</u>.

	First name(s)	Family name (this name will appear in full on the final publication and will be searchable in various abstract and indexing databases)	Affiliated institute	E-mail address
21 1st author	WILLIAM	STEWART	UNI GLASGOW	william.stewart@glasgow.ac.uk
2nd author	KAREN	BLYTH	"	karen.blyth@ "
3rd author	KEVIN	RYAN	"	kevin.ryan@ "
4th author	ANTHONY	CHALMER	"	anthony-chalmers@ "
5th author	JIM	NORMAN	"	jim.norman@glasgow.ac.uk
6th author	STEPHE	TAIT (CA)	"	stephen.tait@glasgow.ac.uk
7th author				
8th author				
9th author				
10th author				

Please use an additional sheet if there are more than 10 authors.

SPRINGER NATURE Springer Nature is one of the world's leading global research, educational and professional publishers, created in May 2015 through the combination of Nature Publishing Group, Palgrave Macmillan, Macmillan Education and Springer Science+Business Media.

SPRINGER NATURE

Change of authorship request form - Journals (pre-acceptance)

Section 6: Declaration of agreement. *All* authors, unchanged, new and removed *must* sign this declaration.
(NB: Please print the form, (docu)-sign and return/upload a scanned copy. Please note that signatures that have been inserted as an image file are acceptable as long as it is handwritten. Typed names in the signature box are unacceptable.) *** Please delete as appropriate. Delete all of the bold if you were on the original authorship list and are remaining as an author.**

	First name	Family name		Signature	Date
1st author	Anna	Koessinger	I agree to the proposed new authorship shown in section 4 **/and the addition/removal*of my name to the authorship list** /and the proposed change in corresponding author		02/04/22
2nd author	Cat	Cloix	I agree to the proposed new authorship shown in section 4 **/and the addition/removal*of my name to the authorship list** /and the proposed change in corresponding author		02/04/22
3rd author	Dom	Koessinger	I agree to the proposed new authorship shown in section 4 **/and the addition/removal*of my name to the authorship list** /and the proposed change in corresponding author		02/04/22
4th authors	Dietar	Heiland	I agree to the proposed new authorship shown in section 4 **/and the addition/removal*of my name to the authorship list** /and the proposed change in corresponding author		02/04/22
5th author	Flo	Bock	I agree to the proposed new authorship shown in section 4 **/and the addition/removal*of my name to the authorship list** /and the proposed change in corresponding author		02/04/22
6th author	Karen	Strathdee	I agree to the proposed new authorship shown in section 4 **/and the addition/removal*of my name to the authorship list** /and the proposed change in corresponding author		02/04/22
7th author	Kev	Kinch	I agree to the proposed new authorship shown in section 4 **/and the addition/removal*of my name to the authorship list** /and the proposed change in corresponding author	Kevin Kinch	02/04/22

SPRINGER NATURE Springer Nature is one of the world's leading global research, educational and professional publishers, created in May 2015 through the combination of Nature Publishing Group, Palgrave Macmillan, Macmillan Education and Springer Science+Business Media.

SPRINGER NATURE

Change of authorship request form - Journals (pre-acceptance)

	First name	Family name		Signature	Date
8th author	Laura	Escardo	I agree to the proposed new authorship shown in section 4 /~~and the addition/removal*of my name to the authorship list~~ /and the proposed change in corresponding author	[signature]	02/04/22
9th author	Nikki	Paul	I agree to the proposed new authorship shown in section 4 /~~and the addition/removal*of my name to the authorship list~~ /and the proposed change in corresponding author	Nikki Paul	02/04/22
10th author	Colin	Nixon	I agree to the proposed new authorship shown in section 4 /~~and the addition/removal*of my name to the authorship list~~ /and the proposed change in corresponding author	[signature]	02/04/22

Please use an additional sheet if there are more than 10 authors.

In case of author collaborations with formal agreement:

	Name of consortium/consortia	First name	Family name		Signature	Date
Representative/legal guarantor				I agree to the proposed new authorship shown in section 4 /**and the addition/removal*of my name to the authorship list** /and the proposed change in corresponding author		

Both added/removed authors should complete the information in the first table under Section 6.

---- End of form ----

SPRINGER NATURE Springer Nature is one of the world's leading global research, educational and professional publishers, created in May 2015 through the combination of Nature Publishing Group, Palgrave Macmillan, Macmillan Education and Springer Science+Business Media.

SPRINGER NATURE

Change of authorship request form - Journals (pre-acceptance)

Section 6: Declaration of agreement. *All* authors, unchanged, new and removed *must* sign this declaration.
(NB: Please print the form, (docu)-sign and return/upload a scanned copy. Please note that signatures that have been inserted as an image file are acceptable as long as it is handwritten. Typed names in the signature box are unacceptable.) *** Please delete as appropriate. Delete all of the bold if you were on the original authorship list and are remaining as an author.**

		First name	Family name		Signature	Date
11	1st author	Gaurav	Malviya	I agree to the proposed new authorship shown in section 4 ~~**/and the addition/removal*of my name to the authorship list**~~ /and the proposed change in corresponding author		02/04/22
12	2nd author	Mark	Jackson	I agree to the proposed new authorship shown in section 4 ~~**/and the addition/removal*of my name to the authorship list**~~ /and the proposed change in corresponding author		02/04/22
13	3rd author	Kirsteen	Campbell	I agree to the proposed new authorship shown in section 4 ~~**/and the addition/removal*of my name to the authorship list**~~ /and the proposed change in corresponding author		02/04/22
14	4th authors	Katrina	Stevenson	I agree to the proposed new authorship shown in section 4 ~~**/and the addition/removal*of my name to the authorship list**~~ /and the proposed change in corresponding author		02/04/22
13	5th author	Sandeep	Davis	I agree to the proposed new authorship shown in section 4 ~~**/and the addition/removal*of my name to the authorship list**~~ /and the proposed change in corresponding author	Type text here	02/04/22
16	6th author	Yassmin	Elmasry	I agree to the proposed new authorship shown in section 4 ~~**/and the addition/removal*of my name to the authorship list**~~ /and the proposed change in corresponding author		02/04/22
17	7th author	Asma	Ahmed	I agree to the proposed new authorship shown in section 4 ~~**/and the addition/removal*of my name to the authorship list**~~ /and the proposed change in corresponding author		02/04/22

SPRINGER NATURE Springer Nature is one of the world's leading global research, educational and professional publishers, created in May 2015 through the combination of Nature Publishing Group, Palgrave Macmillan, Macmillan Education and Springer Science+Business Media.

SPRINGER NATURE Change of authorship request form - Journals (pre-acceptance)

	First name	Family name		Signature	Date
18th author	Jim	Oprey	I agree to the proposed new authorship shown in section 4 **/and ~~the addition/removal*of my name to the authorship~~ list** /and the proposed change in corresponding author	[illegible]	02/04/22
19th author	Gabriel	Ichim	I agree to the proposed new authorship shown in section 4 **/and ~~the addition/removal*of my name to the authorship list~~** /and the proposed change in corresponding author	[illegible]	02/04/22
~~10~~th author 20	Oliver	Schnell	I agree to the proposed new authorship shown in section 4 **/and the addition/~~removal*of my name to the authorship list~~** /and the proposed change in corresponding author	[illegible]	02/04/22

Please use an additional sheet if there are more than 10 authors.

In case of author collaborations with formal agreement:

	Name of consortium/consortia	First name	Family name		Signature	Date
Representative/legal guarantor				I agree to the proposed new authorship shown in section 4 **/and the addition/removal*of my name to the authorship list** /and the proposed change in corresponding author		

Both added/removed authors should complete the information in the first table under Section 6.

---- End of form ----

SPRINGER NATURE Springer Nature is one of the world's leading global research, educational and professional publishers, created in May 2015 through the combination of Nature Publishing Group, Palgrave Macmillan, Macmillan Education and Springer Science+Business Media.

SPRINGER NATURE

Change of authorship request form - Journals (pre-acceptance)

Section 6: Declaration of agreement. *All* authors, unchanged, new and removed *must* sign this declaration.
(NB: Please print the form, (docu)-sign and return/upload a scanned copy. Please note that signatures that have been inserted as an image file are acceptable as long as it is handwritten. Typed names in the signature box are unacceptable.) *** Please delete as appropriate. Delete all of the bold if you were on the original authorship list and are remaining as an author.**

		First name	Family name		Signature	Date
21	1st author	William	Stewart	I agree to the proposed new authorship shown in section 4 /~~**and the addition/removal*of my name to the authorship list**~~ /and the proposed change in corresponding author	[illegible]	02/04/22
22	2nd author	Karen	Blyth	I agree to the proposed new authorship shown in section 4 /~~**and the addition/removal*of my name to the authorship list**~~ /and the proposed change in corresponding author	[illegible]	02/04/22
23	3rd author	Kevin	Ryan	I agree to the proposed new authorship shown in section 4 /~~**and the addition/removal*of my name to the authorship list**~~ /and the proposed change in corresponding author	[illegible]	02/04/22
24	4th authors	Anthony	Chalmers	I agree to the proposed new authorship shown in section 4 /~~**and the addition/removal*of my name to the authorship list**~~ /and the proposed change in corresponding author	AJ Chalmers	02/04/22
25	5th author	Jim	Norman	I agree to the proposed new authorship shown in section 4 /~~**and the addition/removal*of my name to the authorship list**~~ /and the proposed change in corresponding author	[illegible]	02/04/22
26	6th author	Stephen	Tait	I agree to the proposed new authorship shown in section 4 /~~**and the addition/removal*of my name to the authorship list**~~ /and the proposed change in corresponding author	Type text here [illegible]	02/04/22
	7th author			I agree to the proposed new authorship shown in section 4 /~~**and the addition/removal*of my name to the authorship list**~~ /and the proposed change in corresponding author		

SPRINGER NATURE Springer Nature is one of the world's leading global research, educational and professional publishers, created in May 2015 through the combination of Nature Publishing Group, Palgrave Macmillan, Macmillan Education and Springer Science+Business Media.
