# Supplementary material for: Increased apoptotic sensitivity of glioblastoma enables therapeutic targeting by BH3-mimetics
Source: Cell Death Differ. 2022 Apr 26;29(10):2089–104. doi: 10.1038/s41418-022-01001-3 (PMC9525582; doi:10.1038/s41418-022-01001-3)
Supplement: Supplementary file 10 — Author contribution list [file 41418_2022_1001_MOESM10_ESM.pdf]

DECLARATION OF CONTRIBUTIONS TO ARTICLE

ADMC

Manuscript Number:	Journal Name:	
CDD-21-0793R	*Cell Death & Differentiation*	(the 'Journal')

Proposed Title of the Contribution:

Increased apoptotic sensitivity of glioblastoma enables therapeutic targeting by BH3-mimetics (the 'Contribution)

Author(s):

AL Koessinger, C Cloix, D Koessinger, DH Heiland, FJ Bock, K Strathdee, K Kinch, L Martínez-Escardó, NR Paul, C Nixon, G Malviya, MR Jackson, KJ Campbell, K Stevenson, S Davis, Y Elmasry, A Ahmed, J O'Prey, G Ichim, O Schnell, W Stewart, K Blyth, KM Ryan, AJ Chalmers, JC Norman and SWG Tait (the 'Authors')

For all *CDD* articles, each person named as an author in the published version must be able to show he or she has contributed substantially to the article.

Authorship credit should be based on 1) substantial contributions to conception and design, acquisition of data, or analysis and interpretation of data; 2) drafting the article or revising it critically for important intellectual content; and 3) final approval of the version to be published. Authors should meet conditions 1, 2 and 3.

Any person who cannot be shown to have made a substantial contribution to the article cannot be listed as an author in the final version. The name of any person who is deemed to have made a minor contribution can, however, appear in the Acknowledgments section of the article.

Please complete the table below to indicate the contributions of all named authors to the manuscript.

Author Full Name:	Specification of Contribution to the Manuscript:
Katrina Stevenson	provided assistance with the in vivo models
Sandeep Davis	provided assistance with the in vivo models
Yassmin Elmasry	performed and supported data analysis
Asma Ahmed	provided assistance with the in vivo models
Jim O'Prey	performed particular experiments and provided expertise
Gabriel Ichim	expertise and critical input as well as review of the manuscript
Oliver Schnell	expertise and review of the manuscript
William Stewart	provided expertise regarding the human GBM specimens and critical input as well as review of the project
Karen Blyth	PPL holder, overview of in vivo experiments, provided expertise and critical input as well as review of the project
Kevin M Ryan	expertise and critical input as well as review of the project and manuscript
Anthony J Chalmers	PPL holder, overview of in vivo experiments, provided the RNAseq dataset, and GBM specimens, expertise and critical input as well as review of the proect and manuscript
Jim C Norman	expertise and critical input as well as review of the project and manuscript
Stephen WG Tait	conceived the study, designed the work plan and wrote the manuscript

DECLARATION OF CONTRIBUTIONS TO ARTICLE

ADMC

Manuscript Number:	Journal Name:	
	Cell Death & Differentiation	(the 'Journal')

Proposed Title of the Contribution:

	(the 'Contribution)

Author(s):

	(the 'Authors')

For all *CDD* articles, each person named as an author in the published version must be able to show he or she has contributed substantially to the article.

Authorship credit should be based on 1) substantial contributions to conception and design, acquisition of data, or analysis and interpretation of data; 2) drafting the article or revising it critically for important intellectual content; and 3) final approval of the version to be published. Authors should meet conditions 1, 2 and 3.

Any person who cannot be shown to have made a substantial contribution to the article cannot be listed as an author in the final version. The name of any person who is deemed to have made a minor contribution can, however, appear in the Acknowledgments section of the article.

Please complete the table below to indicate the contributions of all named authors to the manuscript.

Author Full Name:	Specification of Contribution to the Manuscript:

Please complete the table below to indicate the contributions of all named authors to the figures.

Figure 1:

Figure 2:

Figure 3:

Figure 4:

Figure 5:

Figure 6:

Signed for and on behalf of the Author(s):	Print Name:	Date:

Please complete the table below to indicate the contributions of all named authors to the figures.

Figure 1:

AL Koessinger and DH Heiland performed and analysed experiments
C Nixon performed IHC staining
AJ Chalmers provided human GBM specimens
MR Jackson analysed clonogenic survival assay and calculated SER in Supp. Figure 1E
D Koessinger provided support with data analysis
C Cloix and K Strathdee performed experiment for Supp. Figure 1F

Figure 2:

AL Koessinger performed and analysed in vitro experiments
K Strathdee performed experiment for Figure 2 B
L Martínez-Escardó performed experiment for Sup. Figure 2A
MR Jackson supported ELDA analysis for Figure 2 B
AL Koessinger, and D Koessinger and K Stevenson performed in vivo experiment
G Malviya and D Koessinger performed animal imaging
AJ Chalmers PPL holder for in vivo experiment

Figure 3:

AL Koessinger performed and analysed in vitro experiments

Figure 4:

AL Koessinger and DH Heiland performed and analysed experiments
FJ Bock and Y Elmasry performed data analysis for Figure 4A

Figure 5:

AL Koessinger performed and analysed experiments
K Kinch and W Stewart supported human GBM specimen collection and analysis
NR Paul acquired images and analysis for Figure 5G
C Nixon performed IHC staining

Figure 6:

AL Koessinger performed and analysed in in vitro and in vivo experiments
J O’Prey supported in vitro experiments
C Cloix performed in vitro experiment in Figure 6A and supported in vivo experiments
D Koessinger, S Davis and A Ahmed supported in vivo experiments
K Blyth PPL holder for in vivo experiment
C Nixon performed IHC staining

Signed for and on behalf of the Author(s):	Print Name:	Date:
